# An Endoplasmic Reticulum CREC Family Protein Regulates the Egress Proteolytic Cascade in Malaria Parasites

**DOI:** 10.1128/mBio.03078-19

**Published:** 2020-02-25

**Authors:** Manuel A. Fierro, Beejan Asady, Carrie F. Brooks, David W. Cobb, Alejandra Villegas, Silvia N. J. Moreno, Vasant Muralidharan

**Affiliations:** a Center for Tropical and Emerging Global Diseases, University of Georgia, Athens, Georgia, USA; b Department of Cellular Biology, University of Georgia, Athens, Georgia, USA; University of Michigan Medical School; Washington University School of Medicine

**Keywords:** calcium-binding protein, malaria, *Plasmodium*, egress, endoplasmic reticulum

## Abstract

The endoplasmic reticulum (ER) is thought to play an essential role during egress of malaria parasites because the ER is assumed to be required for biogenesis and secretion of egress-related organelles. However, no proteins localized to the parasite ER have been shown to play a role in egress of malaria parasites. In this study, we generated conditional mutants of the Plasmodium falciparum endoplasmic reticulum-resident calcium-binding protein (PfERC), a member of the CREC family. Knockdown of the PfERC gene showed that this gene is essential for asexual growth of P. falciparum. Analysis of the intraerythrocytic life cycle revealed that PfERC is essential for parasite egress but is not required for protein trafficking or calcium storage. We found that PfERC knockdown prevents the rupture of the parasitophorous vacuole membrane. This is because PfERC knockdown inhibited the proteolytic maturation of the subtilisin-like serine protease SUB1. Using double mutant parasites, we showed that PfERC is required for the proteolytic maturation of the essential aspartic protease plasmepsin X, which is required for SUB1 cleavage. Further, we showed that processing of substrates downstream of the proteolytic cascade is inhibited by PfERC knockdown. Thus, these data establish that the ER-resident CREC family protein PfERC is a key early regulator of the egress proteolytic cascade of malaria parasites.

## Abstract (precis)

The divergent eukaryotic parasites that cause malaria grow and divide within a vacuole inside a host cell, which they have to break open once they finish cell division. The egress of daughter parasites requires the activation of a proteolytic cascade, and a subtilisin-like protease initiates a proteolytic cascade to break down the membranes blocking egress. It is assumed that the parasite endoplasmic reticulum plays a role in this process, but the proteins in this organelle required for egress remain unknown. We have identified an early ER-resident regulator essential for the maturation of the recently discovered aspartic protease in the egress proteolytic cascade, plasmepsin X, which is required for maturation of the subtilisin-like protease. Conditional loss of PfERC results in the formation of immature and inactive egress proteases that are unable to breakdown the vacuolar membrane barring release of daughter parasites.

## INTRODUCTION

Members of the phylum *Apicomplexa* are responsible for severe human diseases such as malaria, toxoplasmosis, and cryptosporidiosis. Together, the members of this group of obligate intracellular parasites cause several hundred million infections every year and remain among the major drivers of infant mortality (1–4). In fact, malaria results in nearly half a million deaths each year and most of the mortality is attributed to one species, Plasmodium falciparum. All the clinical symptoms of malaria are directly correlated with the asexual expansion of P. falciparum within the host red blood cells (RBCs).

The egress and subsequent invasion of daughter parasites into host cells are essential for the propagation of *Plasmodium* parasites. Upon invading the host cell, the parasites create and reside within a host-derived vacuole called the parasitophorous vacuole (PV). Within this vacuole, the parasites grow and divide into daughter cells, which must egress from the host cell to complete the asexual life cycle. This event depends on the generation of specific organelles that are populated by proteases as well as invasion ligands. These organelles are released via timed exocytosis, which is regulated by signaling pathways dependent on the second messengers, cyclic GMP (cGMP) and calcium (Ca^2+^) (5–9). For example, inhibition of cGMP-dependent protein kinase G (PKG) activity blocks egress (6, 10, 11). Ca^2+^ signaling also induces egress, although it is uncertain whether this pathway works downstream of (7) or synergistically with (6, 12, 13) cGMP signaling. The result of the activity of these signaling pathways is thought to be the exocytosis of egress-related vesicles such as exonemes. Exonemes are populated by proteases such as the aspartic protease plasmepsin X (PMX) and the serine protease subtilisin 1 (SUB1). The exocytosis of mature PMX and SUB1 from exonemes into the PV commits the parasites to egress resulting in the rapid (∼10-min) breakdown of the parasitophorous vacuole membrane (PVM) and the RBC membrane (RBCM) (14, 15).

In malaria parasites, the proteases present in egress-related vesicles require proteolytic cleavage for activation (14, 16, 17). For example, SUB1 undergoes two cleavage events. First, the zymogen undergoes Ca^2+^-dependent autoprocessing in the ER (18, 19) and is then cleaved again by PMX (16, 17). In turn, PMX itself is processed from a 67-kDa zymogen to a 45-kDa active protease (16, 17). Current data suggest that PMX does not undergo autoproteolysis for maturation, as PMX inhibitors seem unable to prevent its cleavage into the 45-kDa form (16, 17). Therefore, this suggests that there are as-yet-unknown factors that regulate the maturation of these key egress proteases.

In this work, we focused on the parasite endoplasmic reticulum (ER) because proteins localized to this organelle are thought to play a key role in egress of daughter merozoites. Their putative functions during this life cycle stage include the biogenesis of specific egress-related organelles, transporting proteins to these organelles, and propagating Ca^2+^ signals essential for egress (20, 21). However, none of the proteins responsible for any these functions during egress of apicomplexan parasites have been identified. One potential candidate is the P. falciparum ER-resident calcium-binding protein PfERC (PF3D7_1108600). In malaria parasites, PfERC is the only protein with identifiable Ca^2+^-binding domains localized to the ER and is capable of binding Ca^2+^ (22). However, the biological function of PfERC is unknown. To address this issue, we used a CRISPR/Cas9-based gene editing approach to generate conditional mutants of PfERC as well as double conditional mutants of PfERC and PMX. These mutants allowed us to determine that this ER-resident protein controls the nested proteolytic cascade in P. falciparum that regulates the egress of malaria parasites from human RBCs.

## RESULTS

### PfERC is a CREC family protein localized in the ER.

**FIG 1:**
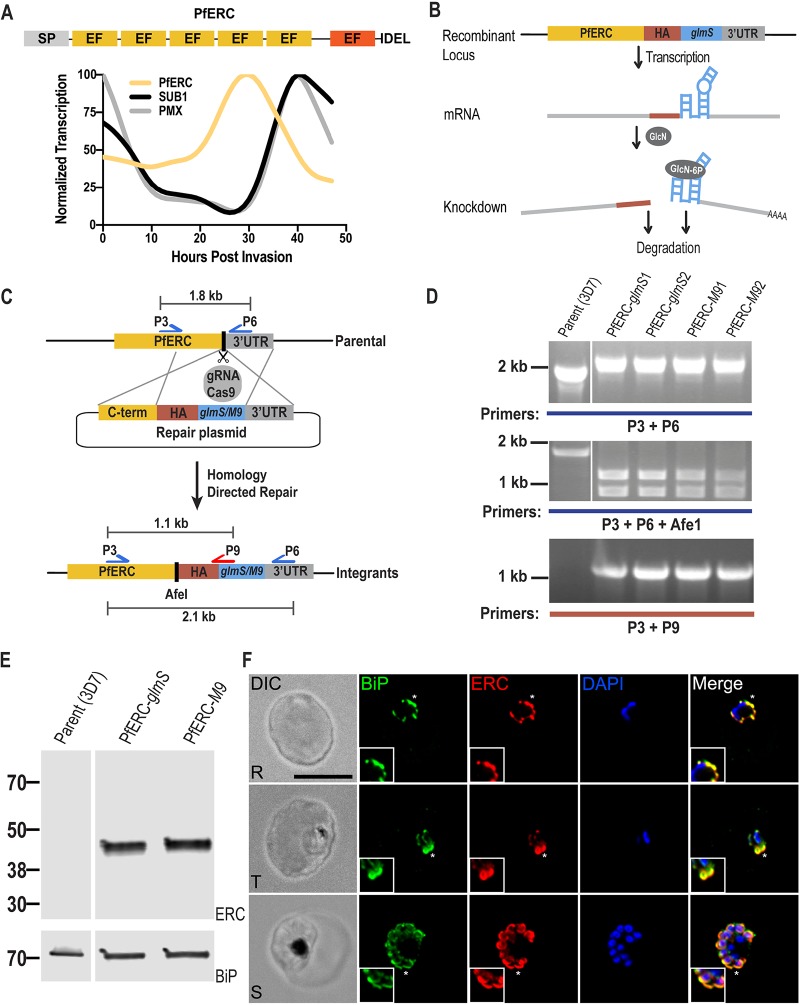
Generation of PfERC-*glmS*/M9 mutant parasites. (A) Schematic representation of the domain structure of PfERC and its transcription profile. PfERC contains a signal peptide, 5 EF hands, and an ER retention signal. There is another degenerate EF hand predicted in PfERC (red box). Transcription data from PlasmoDB.org (26) were normalized to the highest expression value of the total abundance of the transcription. The expression profiles of PfERC and of the egress proteases PMX and SUB1 are shown. (B) Mechanism of PfERC conditional expression using the *glmS* ribozyme system. *glmS* is an inactive ribozyme that is transcribed, but not translated, with the mRNA of a protein of interest. Addition of glucosamine (GlcN) leads to its phosphorylation within the cell to glucosamine-6-phosphate (GlcN-6P). GlcN-6P binds to the transcribed PfERC-*glmS* mRNA, and the *glmS* ribozyme is activated and cleaves itself from the mRNA. This leads to disassociation of the mRNA from its poly(A) tail and results in the degradation of target-specific mRNA. The resulting decline in mRNA levels leads to reduced protein levels and thus to loss of gene expression. As a control, we generated parasite lines containing a mutated version of the *glmS* ribozyme, called *M9*, which cannot cleave itself upon binding of GlcN. (C) Using the CRISPR/Cas9 system and a guide RNA targeting the PfERC gene, we induced a double-stranded break in the PfERC locus that was repaired by a donor plasmid containing templates homologous to the PfERC locus and that appended a C-terminal 3×HA tag, the ER retention signal, and a stop codon followed by the *glmS* or *M9* sequence to the targeted gene. The locations of diagnostic primers (P3, P6, and P9) used to demonstrate the repair of the locus via double-crossover homologous integration are also shown. (D) PCR analysis of the generated mutants using specific primers (P3 and P6; see Table S1 in the supplemental material) in the C terminus and 3′UTR of PfERC shows integration of the HA tag and *glms*/*M9* ribozymes into the PfERC locus. Modification of the PfERC gene introduced an AfeI restriction enzyme site in this locus that was absent in the parental line. Digestion of the PCR products (using AfeI) resulting from amplification performed using primers P3 and P6 shows that AfeI is able to digest the PCR products from our mutants but not those from the parental line. PCR analysis using another primer pair (P3 and P9) that sits on the *glmS*/*M9* sequence shows that amplification occurs only in the mutants and not in the parental line. (E) Western blot of lysates isolated from two independent clones and the parental line (3D7) probed with anti-HA antibodies showed that the PfERC gene was tagged with HA in the mutants but not in the parental line. PfBiP was the loading control. (F) Representative IFA of PfERC-*M9* parasites showing that tagged PfERC localized to the ER as shown with colocalization with the ER chaperone BiP in all asexual stages of the parasite. Phase-contrast (DIC [differential interference contrast]), anti-BiP antibody (green), anti-HA antibody (red), DAPI (blue), and fluorescence merge images are shown from left to right. Images representing colocalization between PfERC and BiP (indicated by asterisks) have been magnified and are shown in the insets. Abbreviations: R, rings; T, trophozoites; S, schizonts. Bar, 5 μm.

PfERC is a protein related to the CREC (calumenin, reticulocalbin 1 and 3, ERC-55, Cab-45) family of proteins, which are characterized by the presence of multiple EF hands and localization in various parts of the secretory pathway (23, 24) (Fig. 1A; see also Fig. S1 in the supplemental material). The domain structure of PfERC is homologous to those of other members of the CREC family of proteins, which include a signal peptide, multiple EF hands, and an ER retention signal (Fig. 1A; see also Fig. S1). However, PfERC differs from its mammalian homologs in that it contains only 5 predicted EF hands, although a sixth degenerate EF hand (residues 314 to 325) may be present in its extended C terminus (Fig. 1A; see also Fig. S1) (22). Various roles, including Ca^2+^ signaling and homeostasis, have been attributed to CREC members, and one member, RCN3, has been shown to interact with the subtilisin-like peptidase, PACE4, though the functional significance of this interaction is unknown (23, 25). As the level of PfERC expression peaks in early schizont stage parasites, we hypothesized that PfERC is required for egress of daughter parasites during this terminal stage of the asexual life cycle (22, 26) (Fig. 1A).

10.1128/mBio.03078-19.1 FIG S1 Sequence alignment of PfERC to other members of the CREC family of proteins by the use of MUSCLE alignment, viewed using JalView software (http://www.jalview.org/) and BOXSHADE (65). Alignment was done using the following human homologs: Cab-45, ERC-55, reticulocalbin 1 (RCN1), reticulocalbin 3 (RCN3), and calumenin. Identical residues are shaded in black, similar residues are shaded in gray, and EF hands are highlighted in red. Download FIG S1, PDF file, 1.0 MB. Copyright © 2020 Fierro et al. 2020 Fierro et al. This content is distributed under the terms of the Creative Commons Attribution 4.0 International license.

10.1128/mBio.03078-19.10 TABLE S1 Primers used in this study. Download Table S1, DOCX file, 0.01 MB. Copyright © 2020 Fierro et al. 2020 Fierro et al. This content is distributed under the terms of the Creative Commons Attribution 4.0 International license.

### Generating conditional mutants of PfERC.

In order to determine the biological role of PfERC, we used CRISPR/Cas9 gene editing to generate conditional mutants of PfERC. In these parasite lines, the endogenous locus of PfERC was tagged with the inducible version of the ribozyme gene, *glmS*, or with the inactive version of the ribozyme, *M9* (termed PfERC-*glmS* and PfERC-*M9*, respectively) (Fig. 1B) (27). PCR analysis of DNA isolated from PfERC-*glmS* and PfERC-*M9* parasite clones from two independent transfections demonstrated the correct insertion of the hemagglutinin (HA) tag, an ER retention signal, and the *glmS*/*M9* ribozyme genes at the endogenous PfERC locus (Fig. 1C and D). These independently isolated PfERC-*glmS* and PfERC-*M9* parasite clones were utilized in all subsequent experiments. We detected expression of PfERC fused to the HA tag in the PfERC-*glmS* and PfERC-*M9* clones at the expected size but not in the parental line (Fig. 1E). Immunofluorescence microscopy showed that PfERC colocalized with the ER marker, PfBiP (Fig. 1F). Similar colocalization has been observed for several other *Plasmodium* ER proteins such as plasmepsin V, signal peptidase complex 25, signal peptidase complex 21 (28), and glucose-responsive protein 170 (29).

**FIG 2:**
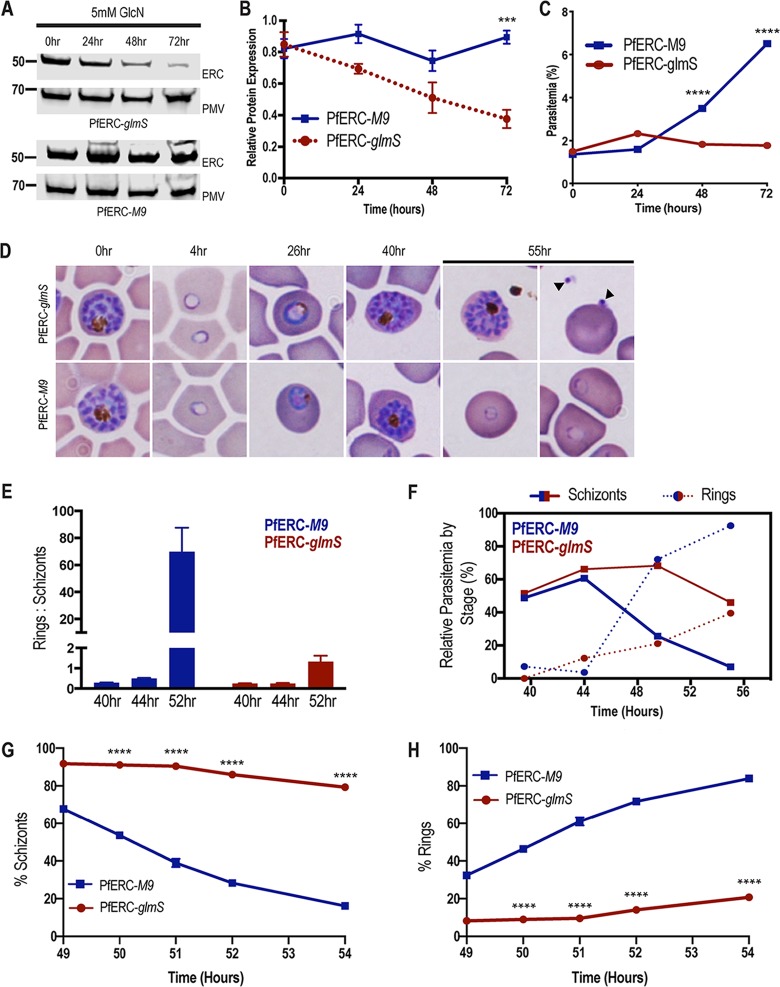
PfERC mutants fail to transition from schizonts to rings. (A) Western blot of parasite lysates isolated from PfERC-*glmS* and PfERC-*M9* parasites grown in the presence of 5 mM GlcN and probed with anti-HA antibodies and anti-plasmepsin V (PMV) antibodies. Results of one representative experiment of four are shown. (B) Quantification of changes in expression of PfERC in PfERC-*glmS* and PfERC-*M9* parasites after addition of GlcN, as described for panel A. Data were normalized to the loading control (PMV) and are shown as means ± standard errors of the means (*n* = 4 biological replicates; ***, *P* < 0.001 [2-way analysis of variance {ANOVA}]). (C) Growth of asynchronous PfERC-*glmS* and PfERC-*M9* parasites incubated with 5 mM GlcN, over 4 days, was observed using flow cytometry. Data are presented as means ± standard errors of the means (*n* = 3 technical replicates; ****, *P* < 0.0001 [2-way ANOVA]). One representative data set from 4 biological replicates is shown (Fig. S2A). (D) Representative Hema-3-stained blood smears of synchronous PfERC-*glmS* and PfERC-*M9* parasites grown in the presence of GlcN (*n* = 2 biological replicates). (E) GlcN was added to synchronous PfERC-*glmS* and PfERC-*M9* schizonts, and parasite stages were determined using flow cytometry. The ratio of rings to schizonts was calculated using the number of rings and schizonts observed at each time point. Data are presented as means ± standard errors of the means. (F) Hema-3-stained blood smears of synchronous PfERC-*glmS* and PfERC-*M9* parasites grown in the presence of GlcN (shown in panel D) were manually counted. The amount of each life cycle stage (ring, trophozoite, and schizont) was determined as a percentage of the total number of parasites for each time point. (G and H) GlcN was added to synchronous PfERC-*glmS* and PfERC-*M9* schizonts, and parasite stages were determined for schizonts (G) and rings (H) by the use of flow cytometry. At each time point, cells were fixed and stained with the DNA dye Hoescht 33342 to distinguish between ring stage parasites (1 N) and schizont stage parasites (16 to 32 N). Results of one representative experiment of three biological replicates are shown. Data are presented as means ± standard errors of the means (*n* = 3 technical replicates; ****, *P* < 0.0001 [2-way ANOVA]).

To determine whether PfERC is essential for intraerythrocytic survival, we grew asynchronous PfERC-*glmS* and PfERC-*M9* parasites in the presence of glucosamine (GlcN), which activates the *glmS* ribozyme, leading to mRNA cleavage (Fig. 1B). We observed reproducible reduction of PfERC expression in PfERC-*glmS* parasites, while there was no reduction in PfERC expression in PfERC-*M9* parasites grown under identical conditions (Fig. 2A and B; see also Fig. S2A). Importantly, this reduction in PfERC levels inhibited the asexual expansion of PfERC-*glmS* parasites, while the PfERC-*M9* parasites were able to grow normally under the same conditions (Fig. 2C; see also Fig. S2B). This inhibition of the asexual growth of PfERC-*glmS* parasites occurred in a GlcN dose-dependent manner (Fig. S2C).

10.1128/mBio.03078-19.2 FIG S2 Effect of PfERC knockdown on parasite growth. (A) Representative image of results of Western blotting of lysates from PfERC-*glmS* and PfERC-*M9* as described in the Fig. 2A legend. (B) One growth curve (representing four biological replicates) of PfERC clones grown in the presence of 5 mM GlcN. Data are presented as means ± standard errors of the means. (C) Asynchronous PfERC-*glmS* parasites were incubated in different concentrations of GlcN, and growth after three days was assessed by flow cytometry. Data are presented as means ± standard errors of the means of results from *n* = 2 biological replicates. (D) Levels of rings (red), trophozoites (black), and schizonts (blue) as percentages of total parasites as scored by light microscopy of Hema-3-stained blood smears from synchronous PfERC-*glmS* and PfERC-*M9* parasites grown in the presence of GlcN (*n* = 2 biological replicates). Download FIG S2, PDF file, 0.4 MB. Copyright © 2020 Fierro et al. 2020 Fierro et al. This content is distributed under the terms of the Creative Commons Attribution 4.0 International license.

### PfERC is essential for schizont to ring transition.

Since our data showed that PfERC was essential for growth within the host RBC, we used synchronous parasites to determine which asexual stage was affected by knockdown. We added GlcN to synchronized schizonts and observed the morphological development of the asexual stages at regular intervals during the intraerythrocytic life cycle (Fig. 2D). All intracellular stages were morphologically normal in both PfERC-*glmS* and PfERC-*M9* parasites grown with GlcN (Fig. 2D; see also Fig. S2D). However, 55 h after addition of GlcN, the PfERC-*glmS* parasites either remained as morphologically normal schizonts or were observed as daughter merozoites in the extracellular space and were also observed attached to RBCs (Fig. 2D). On the other hand, PfERC-*M9* parasites were able to egress and reinvade fresh RBCs and developed into ring stage parasites (Fig. 2D; see also Fig. S2D).

These data suggest that knockdown of PfERC resulted in a defect in the conversion of schizonts into rings. To test this, we induced knockdown and observed the conversion of schizonts into rings via flow cytometry at 44, 48, and 56 h after addition of GlcN. We found that over the course of 12 h, PfERC-*M9* parasites transitioned from schizonts to rings as determined by the ring/schizont ratio whereas PfERC-*glmS* parasites were unable to convert from schizonts into rings, resulting in a drastically reduced ratio (Fig. 2E). Using synchronized PfERC-*glmS* and PfERC-*M9* parasites, treated with GlcN as described above, we observed the final hours of the asexual life cycle using thin blood smears and quantified the parasites using flow cytometry (Fig. 2F to H; see also Fig. S2D). These data show that there was a delay in the disappearance of the morphologically normal PfERC-*glmS* schizonts over the final few hours of the asexual life cycle compared to results seen with the PfERC-*M9* schizonts, suggesting that knockdown of PfERC led to a defect in egress (Fig. 2F and G). Consequently, the delayed egress led to reduced numbers of ring stage parasites in PfERC-*glmS* parasites in comparison to PfERC-*M9* parasites (Fig. 2F and H).

### PfERC is required for PVM breakdown.

**FIG 3:**
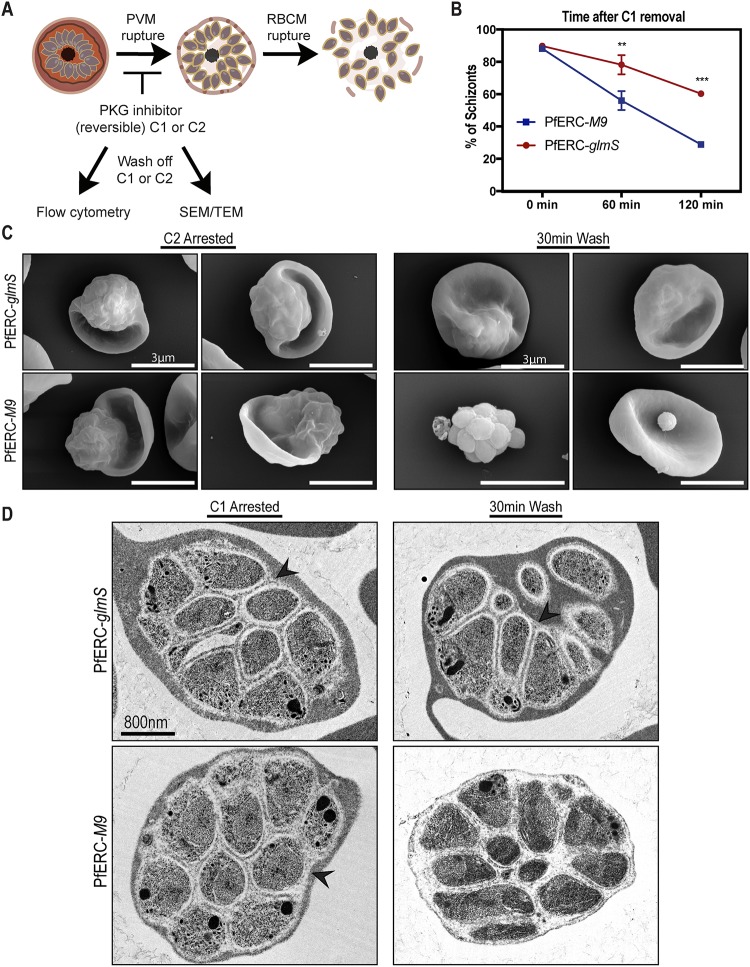
PfERC knockdown inhibits PVM breakdown. (A) Schematic showing the experimental layout used to study the effect of PfERC knockdown using specific compounds that inhibit egress in parasites. Abbreviations: C1 and C2, compound 1 and compound (2 PKG inhibitors); SEM, scanning electron microscopy; TEM, transmission electron microscopy. (B) As described for panel A, synchronized PfERC-*glmS* and PfERC-*M9* schizonts were grown in the presence of GlcN and second-cycle schizonts were observed by flow cytometry after removal of C1 (time 0 h). Schizonts were quantified as a percentage of the total amount of parasites as determined by flow cytometry. Data are presented as means ± standard errors of the means (*n* = 3 biological replicates; **, *P* < 0.01; ***, *P* < 0.001 [2-way ANOVA]). (C) Representative SEM images of C2-arrested PfERC-*glmS* (*n* = 4 biological replicates) and PfERC-*M9* (*n* = 4 biological replicates) mutants fixed immediately after washing off C2 and after 30 min as described for panel A. (D) Representative TEM images of PfERC-*glmS* (*n* = 2 biological replicates) and PfERC-*M9* (*n* = 2 biological replicates) schizonts incubated with compound 1 as described for panel A. Bar, 800 nm. PVM is marked by black arrowheads.

Egress of daughter merozoites from the infected RBCs is an ordered and rapid process where the PVM breakdown precedes the disruption of the RBCM (Fig. 3A) (11). Therefore, we took synchronized PfERC-*glmS* and PfERC-*M9* schizonts and initiated knockdown by addition of GlcN. These schizonts were allowed to reinvade fresh RBCs and to proceed through the asexual stages for 48 h until they developed into schizonts again. Then, these second-cycle schizonts were incubated with inhibitors that block key steps during egress of P. falciparum (Fig. 3A). To ensure synchronized egress, we used reversible inhibitors of PKG, namely, compound 1 (C1) {4-[2-(4-fluorophenyl)-5-(1-methylpiperidine-4-yl)-1H-pyrrol-3-yl] pyridine} and compound 2 (C2) {4-[7- [(dimethylamino)methyl]-2-(4-fluorphenyl)imidazo[1,2-a]pyridine-3-yl]pyrimidin-2-amine}, because inhibition of PKG allows merozoites to develop normally but prevents them from initiating egress (Fig. 3A) (6, 11). We used flow cytometry to observe PfERC-*glmS* and PfERC-*M9* schizonts after washing off C1 and saw that there was a delay in the egress of PfERC-*glmS* schizonts whereas the majority (>60%) of the PfERC-*M9* schizonts were able to complete egress within 2 h after washout of C1 (Fig. 3B). Consequently, this led to a loss of ring formation in PfERC-*glmS* parasites compared to PfERC-*M9* parasites (Fig. S3A). Removal of C1 initiated the breakdown of the PVM followed by RBCM rupture (Fig. 3A), suggesting that the PfERC-*glmS* parasites failed to breach one of these membranes despite removal of the PKG inhibitor.

10.1128/mBio.03078-19.3 FIG S3 Quantification of C1-treated parasites. (A) As shown in Fig. 3A, synchronized PfERC-*glmS* and PfERC-*M9* schizonts were grown in the presence of GlcN and second-cycle rings were observed by flow cytometry after removal of C1 (time 0 h). Rings were quantified as percentages of the total amount of parasites as determined by flow cytometry. Data are presented as means ± standard errors of the means (*n* = 3 biological replicates; **, *P* < 0.01; ***, *P* < 0.001 [2-way analysis of variance {ANOVA}]). (B) PfERC-*glmS* and PfERC-*M9* schizonts were treated as described in the Fig. 3C legend, and wide-field (10 fields per biological replicate) SEM images were quantified. The collapsed schizonts shown in Fig. 3C were normalized to the total number of schizonts counted in the fields. Data are presented as means ± standard errors of the means (*n* = 4 biological replicates; n.s., nonsignificant; *, *P < *0.05 [one-way ANOVA]). (C) TEM images of C1-treated PfERC mutants. Additional TEM images of C1-treated schizonts of PfERC-glmS and PfERC-M9 parasites after knockdown show normal schizogony as well as organelle biogenesis occurring in our mutants under knockdown conditions. Download FIG S3, PDF file, 0.1 MB. Copyright © 2020 Fierro et al. 2020 Fierro et al. This content is distributed under the terms of the Creative Commons Attribution 4.0 International license.

Therefore, we tested whether PfERC knockdown prevented rupture of PVM or whether PfERC was required for RBCM breakdown (Fig. 3A). PfERC-*glmS* and PfERC-*M9* schizonts (where knockdown had been initiated in the previous cycle) were incubated with C2 (6, 11) and observed by scanning electron microscopy (SEM) (Fig. 3A and C). We observed that the parasites treated with C2 were morphologically identical and had developed into mature schizonts within the RBCs (Fig. 3C). Then, we washed C2 from the parasites and observed these schizonts after 30 min by SEM (Fig. 3C). During this time period, PfERC-*M9* schizonts were able to initiate egress after removal of C2. We observed free PfERC-*M9* merozoites attached to the RBCs as well as clusters of merozoites that had broken out of the PVM but appeared to be contained by a collapsed RBCM wrapped around them (Fig. 3C; see also Fig. S3B). In contrast, the PfERC-*glmS* schizonts were still stuck within the RBC and looked identical to the C2-arrested schizonts, suggesting that they had not initiated egress even though the PKG activity was no longer inhibited (Fig. 3C; see also Fig. S3). These data suggest that knockdown of PfERC blocks egress at an early step, perhaps blocking the rupture of the PVM (Fig. 3C).

We directly observed whether breakdown of the PVM was impacted by knockdown of PfERC by the use of transmission electron microscopy (TEM) (Fig. 3A and D). Similarly to the previous experiment, after GlcN had been added to PfERC-*glmS* and PfERC-*M9* schizonts, the parasites were allowed to go through one asexual cycle. These second-cycle schizonts were prevented from initiating egress by incubation of with C1 (Fig. 3A) and were observed by TEM (Fig. 3D). These PfERC-*glmS* and PfERC-*M9* schizonts were morphologically identical and, as expected, had formed merozoites within the PVM and RBCM (Fig. 3D; see also Fig. S3C). As described above, C1 was washed off these schizonts and the schizonts were observed by the use of TEM after 30 min (Fig. 3D). Our results show that the PfERC-*M9* schizonts were able to break down the PVM within 30 min after removal of C1 whereas the PfERC-*glmS* mutants failed to rupture the PVM (Fig. 3D). Overall, these data demonstrate that PfERC function was critical for the breakdown of the PVM (Fig. 3C and D).

### PfERC is not required for calcium storage.

Since PfERC resides in the ER and possesses Ca^2+^-binding domains, we hypothesized that PfERC is required for egress because it plays a role in Ca^2+^ homeostasis in the ER. To test this model, synchronized PfERC-*glmS* and PfERC-*M9* schizonts were incubated with GlcN and allowed to proceed through one asexual cycle until they again formed schizonts. The second-cycle schizonts were isolated using saponin lysis and loaded with Fluo-4AM to measure cytosolic Ca^2+^ levels (Fig. S4A and B). The health of these saponin-released schizonts was assessed by observing hemozoin crystal dynamics in the digestive vacuole via live microscopy (30).To assess whether the storage of Ca^2+^ in the ER of the parasite was affected by knockdown of PfERC, we added the sarcoplasmic/endoplasmic reticulum Ca^2+^-ATPase (SERCA) inhibitor cyclopiazonic acid (CPA) to these saponin-isolated parasites (Fig. S4A and C) (31). Inhibition of SERCA allows Ca^2+^ stored in the ER to leak into the cytoplasm, which results in a detectable change in the fluorescence of Fluo-4AM (Fig. S4C). Our measurements showed that there were no differences between PfERC-*glmS* and PfERC-*M9* schizonts in the amounts of Ca^2+^ that leaked from the parasite ER, upon SERCA inhibition (Fig. S4C).

10.1128/mBio.03078-19.4 FIG S4 PfERC knockdown is not required for ER Ca^2+^ storage. (A) Experimental schematic showing how Ca^2+^ measurements were done in PfERC-*glmS* and PfERC-*M9* mutants. Synchronized PfERC-*glmS* and PfERC-*M9* schizonts were incubated with GlcN for 48 h and isolated using saponin lysis, which lyses the RBC membrane but leaves the PV intact. CPA, cyclopiazonic acid. (B) Microscopy of Fluo-4AM-treated parasites. Live imaging of representative saponin-purified parasites incubated with Fluo-4AM was performed. No localization of the dye was observed in the food vacuole. Bar, 5 μm. (C) Representative fluorescence tracings after addition of CPA or dimethyl sulfoxide (DMSO) vehicle control to PfERC-*glmS* and PfERC-*M9* schizonts, isolated as described in the panel A legend. Quantification was done by calculating the difference between the basal fluorescence level and the highest peak of fluorescence. Data are represented as combined means ± standard errors of the means (for PfERC-*glmS*, *n* = 15 biological replicates; for PfERC-*M9*, *n* = 9 biological replicates; n.s., nonsignificant; unpaired *t* [test]). (D) Representative fluorescence tracings after addition of ionomycin or DMSO vehicle control to PfERC-*glmS* and PfERC-*M9* schizonts, isolated as described in the panel A legend. Quantification was done by calculating the difference between the basal fluorescence level and the highest peak of fluorescence. Data are represented as combined means ± standard errors of the means (for PfERC-*glmS*, *n* = 9 biological replicates; for PfERC-*M9*, *n* = 5 biological replicates; n.s., nonsignificant; unpaired *t* test). Arrows indicate the times at which a reagent was added. Download FIG S4, PDF file, 0.8 MB. Copyright © 2020 Fierro et al. 2020 Fierro et al. This content is distributed under the terms of the Creative Commons Attribution 4.0 International license.

10.1128/mBio.03078-19.5 FIG S5 IFA of EBA-175 in PfERC mutants. Representative SIM images of parasites grown in the presence of GlcN for 48 h and then incubated with compound 1 for 4 h. Compound 1 was then removed, and the parasites were incubated further with E-64 for 6 h and stained with anti-EBA175 antibodies as well as the nuclear stain. *n* = 2 biological replicates. From top to bottom, the images represent DAPI (blue), anti-EBA175 (green), and fluorescence merge. Bar, 2 μm. Download FIG S5, PDF file, 1.3 MB. Copyright © 2020 Fierro et al. 2020 Fierro et al. This content is distributed under the terms of the Creative Commons Attribution 4.0 International license.

To test if there was a defect in Ca^2+^ storage in neutral stores, we used the ionophore ionomycin, which releases Ca^2+^ from all neutral stores in the cell, and measured the release of Ca^2+^ into the cytoplasm of PfERC-*glmS* and PfERC-*M9* schizonts. The parasites were isolated as described above, and the changes in cytoplasmic Ca^2+^ levels were measured using Fluo-4AM (Fig. S4A and D). Again, we did not observe any differences in the amounts of Ca^2+^ released into the cytoplasm of PfERC-*glmS* and PfERC-*M9* schizonts treated with ionomycin (Fig. S4D). These data suggest that the availability of free Ca^2+^ (or of other neutral Ca^2+^ stores) in the ER of P. falciparum does not change upon knockdown of PfERC. Furthermore, these data suggest that the observed egress defect seen upon PfERC knockdown was not a result of Ca^2+^disequilibrium in the parasite ER.

### PfERC is not required for protein trafficking or organelle biogenesis.

**FIG 4:**
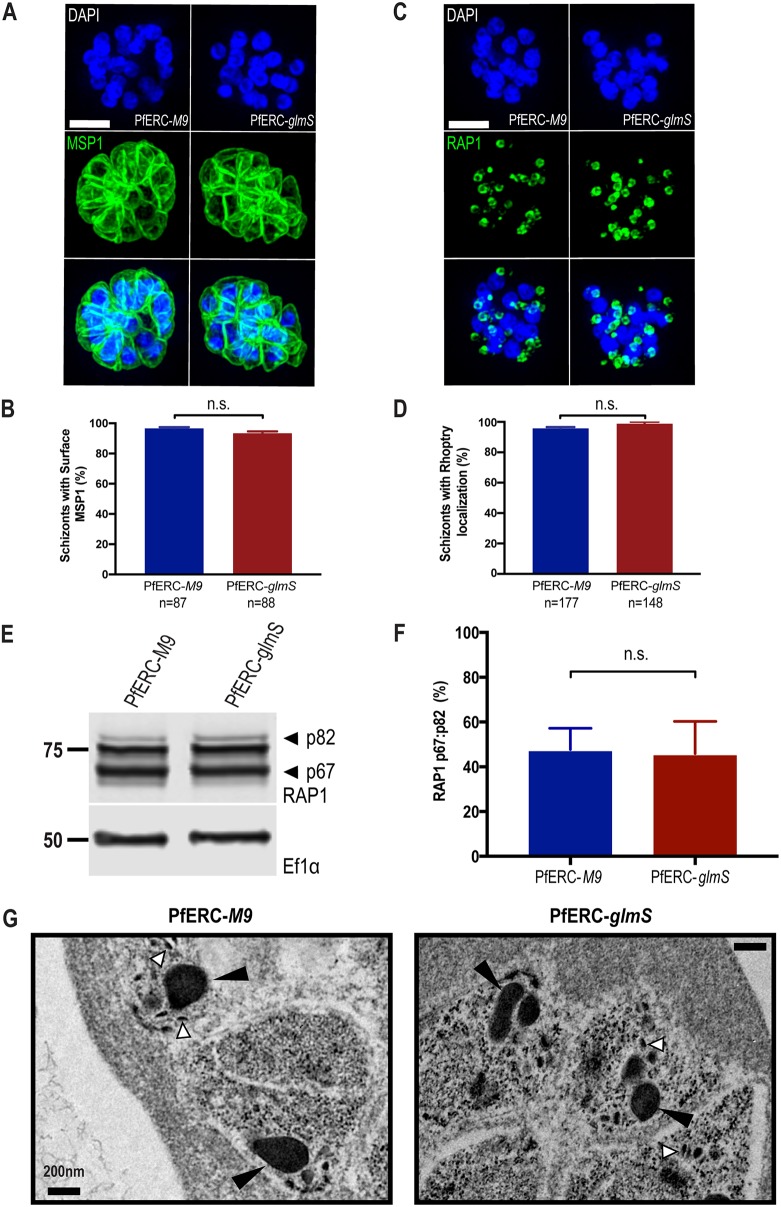
PfERC is not required for protein trafficking or organellar biogenesis. (A) Representative SIM images of PfERC-*glmS* and PfERC-*M9* schizonts incubated with GlcN for 48 h and stained with anti-MSP1 and the nuclear stain DAPI. From top to bottom, the images are DAPI (blue), anti-MSP1 (green), and fluorescence merge. Bar, 2 μm. (B) The surface localization of MSP1 was quantified in PfERC-*glmS* and PfERC-*M9* schizonts incubated with GlcN for 48 h and stained as described for panel A. Data are presented as means ± standard errors of the means (*n* = 2 biological replicates; n.s., nonsignificant; unpaired *t* test). (C) Representative SIM images of PfERC-*glmS* and PfERC-*M9* schizonts incubated with GlcN for 48 h and stained with anti-RAP1 and the nuclear stain DAPI. From top to bottom, the images represent DAPI (blue), anti-RAP1 (green), and fluorescence merge. Bar, 2 μm. (D) The rhoptry localization of RAP1 was quantified in PfERC-*glmS* and PfERC-*M9* schizonts incubated with GlcN for 48 h and stained as described for panel C. Data are presented as means ± standard errors of the means (*n* = 3 biological replicates; n.s., nonsignificant; unpaired *t* test). (E) Western blot of parasite lysates isolated from PfERC-*glmS* and PfERC-*M9* schizonts grown in the presence of GlcN for 48 h and probed with anti-RAP1 (top panel) and anti-EF1α (loading control; bottom panel). Results of one representative experiment of five are shown. The sizes of the marker proteins that comigrated with the probed protein are indicated on the left. (F) Quantification of RAP1 processing in PfERC-*glmS* and PfERC-*M9* parasites incubated with GlcN as described for panel E. Data were normalized to the ratio of processed RAP1 (p67/p82) of PfERC-*M9* parasites and are presented as means ± standard errors of the means (*n* = 5 biological replicates; n.s , nonsignificant; unpaired *t* test). (G) Representative TEM images of synchronized PfERC-*glmS* and PfERC-*M9* schizonts grown for 48 h with GlcN and incubated with C1 for 4 h, as shown in Fig. 3A (*n* = 2 biological replicates). Small arrowheads (white) point to micronemes, while large arrowheads (black) point to rhoptries. Bar, 200 nm.

Several proteins required for egress are synthesized in the ER and trafficked to specific organelles such as exonemes. It is possible that PfERC functions in protein trafficking through the ER and that it may be required for trafficking proteins essential for egress. Therefore, we tested the effect of PfERC knockdown on the localization of several proteins at specific intracellular locations. We tested the localization of merozoite surface protein 1 (MSP1), which is a glycosylphosphatidylinositol (GPI)-anchored merozoite protein secreted to the merozoite plasma membrane (32). Using structured illumination microscopy (SIM), we observed whether GPI-anchored protein MSP1, normally present on the merozoite plasma membrane, was mislocalized upon knockdown of PfERC (Fig. 4A). As described above, knockdown was initiated in synchronized PfERC-*glmS* and PfERC-*M9* schizonts, and after 48 h, these schizonts were stained with anti-MSP1 antibodies. Our data show that there were no differences between developing PfERC-*glmS* and PfERC-*M9* merozoites with respect to trafficking of MSP1 to the surface after knockdown (Fig. 4A and B). Similarly, we observed erythrocyte binding antigen 175 (EBA-175), which localizes to the micronemes, and found that PfERC knockdown did not inhibit the trafficking of EBA-175 to micronemes (Fig. S5).

We then observed the localization of rhoptry-associated protein 1 (RAP1), which also traffics through the ER to the rhoptries. Our data show that there were no differences between PfERC-*glmS* and PfERC-*M9* schizonts in the localization of RAP1 to rhoptries (Fig. 4C and D). After transport to the rhoptry, rhoptry bulb protein RAP1 undergoes essential proteolytic cleavage by the aspartic protease plasmepsin IX (PMIX) from a proform (p83) to a mature form (p67) (16, 17, 33, 34). Therefore, we tested whether RAP1 processing was inhibited by knockdown of PfERC using Western blotting (Fig. 4E; see also Fig. S6A). Our data show that the proteolytic processing of RAP1 was not inhibited by the knockdown of PfERC (Fig. 4E and F) and that knockdown did not lead to a defect in trafficking or processing of proteins in the rhoptries. Together, these data show that knockdown of PfERC does not cause a generalized defect in the secretory pathway (Fig. 4A to F).

**FIG 5:**
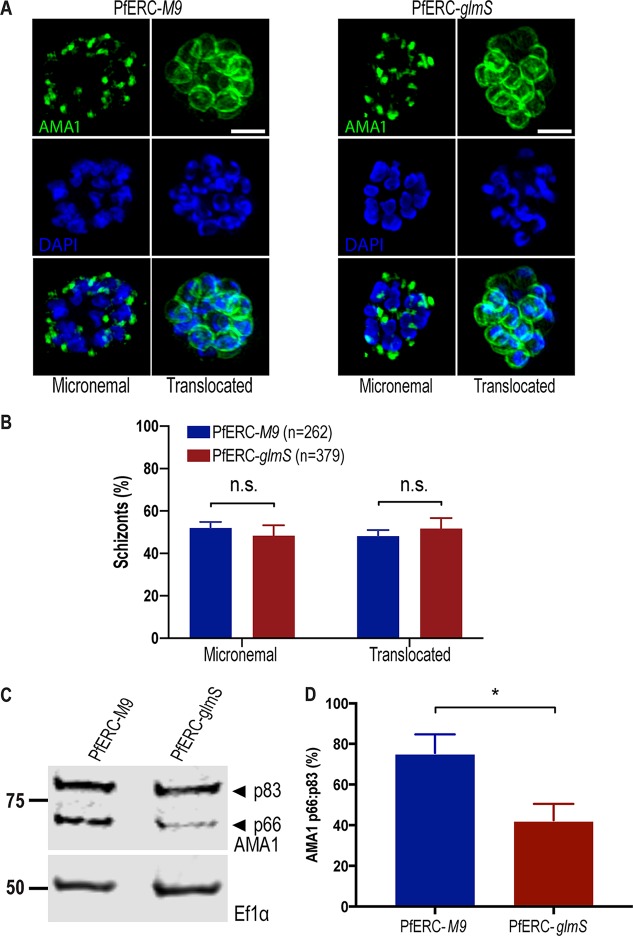
Proteolytic processing of AMA1 requires PfERC. (A) Parasites were grown in the presence of GlcN for 48 h and then incubated with compound 1 for 4 h. Compound 1 was then removed, and parasites were incubated further with E-64 for 6 h and stained with anti-AMA1 as well as the nuclear stain DAPI. Representative SIM images of these PfERC-*glmS* and PfERC-*M9* schizonts are shown. From top to bottom, the images are anti-AMA1 (green), DAPI (blue), and fluorescence merge. Bar, 2 μm. (B) Micronemal and surface (or translocated) localization of AMA1 was quantified in PfERC-*glmS* and PfERC-*M9* schizonts as described for panel A. Data are presented as means ± standard errors of the means (*n* = 4 biological replicates; n.s., nonsignificant; one-way ANOVA). (C) Western blot of parasite lysates isolated from PfERC-*glmS* and PfERC-*M9* schizonts grown in the presence of GlcN for 48 h and probed with anti-AMA1 antibodies (top panel) and anti-EF1α antibodies (loading control; bottom panel). Results of one representative experiment of eight are shown. The sizes of the marker proteins that comigrated with the probed protein are indicated on the left. (D) Quantification of AMA1 maturation in PfERC-*glmS* and PfERC-*M9* parasites incubated with GlcN as described for panel C. Data were normalized to the ratio of processed AMA1 (p66/p83) in PfERC-*M9* parasites and are presented as means ± standard errors of the means (*n* = 8 biological replicates; *, *P* < 0.05 [unpaired *t* test]).

**FIG 6:**
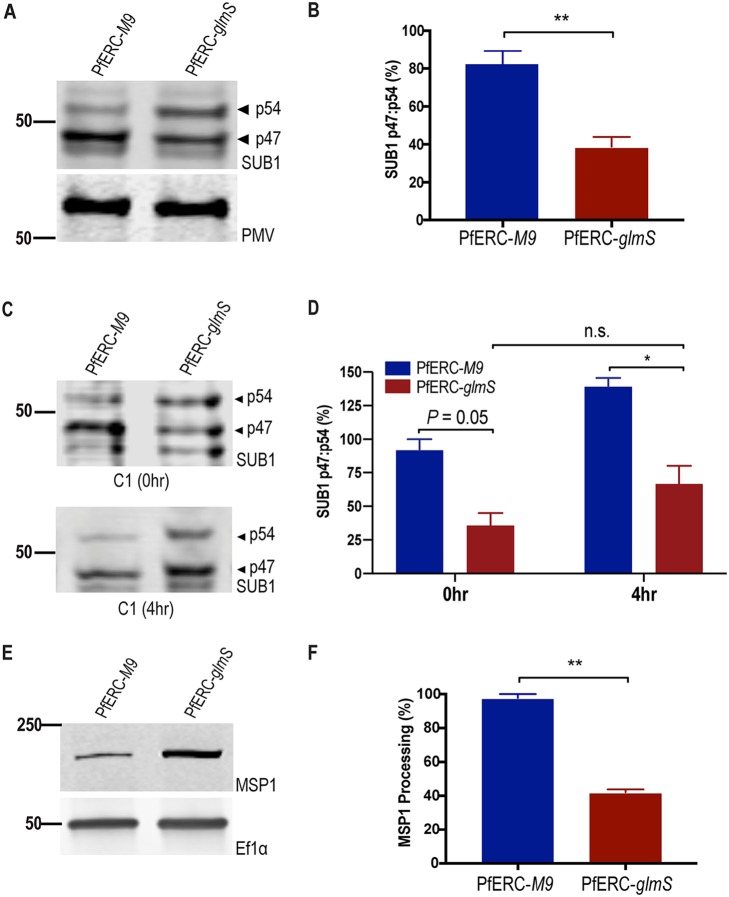
PfERC knockdown inhibits SUB1 and MSP1 processing. (A) Western blot of parasite lysates isolated from PfERC-*glmS* and PfERC-*M9* schizonts grown in the presence of GlcN for 48 h and probed with anti-SUB1 antibodies (top panel) and anti-PMV (loading control; bottom panel). Results of one representative experiment of four are shown. The sizes of the marker proteins that comigrated with the probed protein are indicated on the left. (B) Quantification of SUB1 processing in PfERC-*glmS* and PfERC-*M9* parasites over time after addition of GlcN as described for panel A. Data were normalized to the ratio of processed SUB1 (p47/p54) of PfERC-*M9* parasites and are presented as means ± standard errors of the means (*n* = 4 biological replicates; **, *P* < 0.005 [unpaired *t* test]). (C) Western blot of parasite lysates isolated from PfERC-*glmS* and PfERC-*M9* schizonts grown in the presence of GlcN for 48 h and then incubated with compound 1. Samples were taken either 0 h or 4 h after addition of compound 1. (D) Quantification of SUB1 processing in PfERC-*glmS* and PfERC-*M9* parasites incubated with compound 1 as described for panel C. Data were normalized to the ratio of processed SUB1 (p47/p54) of PfERC-*M9* parasites at 0 h and presented as ± standard errors of the means. (*n* = 2 biological replicates; *, *P < *0.05 [one-way ANOVA]). (E) Western blot of parasite lysates isolated from PfERC-*glmS* and PfERC-*M9* schizonts grown in the presence of GlcN for 48 h and probed with anti-MSP1 12.4 antibodies (top panel) and with anti-EF1α (loading control; bottom panel). Results of one representative experiment of two are shown. (F) Quantification of unprocessed (or full-length) MSP1 in PfERC-*glmS* and PfERC-*M9* parasites after addition of GlcN as described for panel E. Data were normalized to the loading control (EF1α) and are presented as means ± standard errors of the means (*n* = 2 biological replicates; **, *P* < 0.005 [unpaired *t* test]).

**FIG 7:**
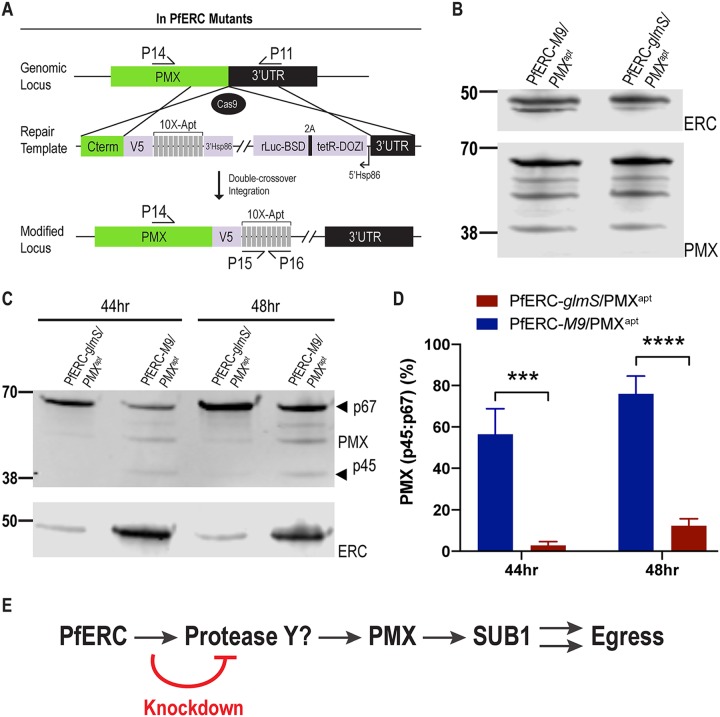
PfERC is required for PMX cleavage. (A) In experiments using a guide RNA targeting the PMX gene in PfERC-*glmS* and PfERC-*M9* mutants, Cas9 generated a double-stranded break in the PMX locus that was repaired by a donor plasmid containing templates homologous to the PMX locus. The homology-directed repair appended a C-terminal 3×-V5 tag and a stop codon followed by 10× *tetR* aptamers to the PMX gene. The locations of the diagnostic primers used to demonstrate repair of the locus via double-crossover homologous integration are also shown (Table S1). (B) Representative Western blots of lysates isolated from asynchronous PfERC-*glmS*/PMX^apt^ and PfERC-*M9*/PMX^apt^ double mutants probed with anti-V5 antibodies show that the PMX gene was tagged with V5 in the double mutants. (C) Representative Western blot of lysates isolated from PfERC-*glmS*/PMX^apt^ and PfERC-*M9*/PMX^apt^ schizonts grown in the presence of GlcN for 44 h (*n* = 6 biological replicates) and 48 h (*n* = 11 biological replicates) and probed with anti-V5 (top panel), anti-HA (bottom panel). (D) Quantification of PMX processing in PfERC-*glmS*/PMX^apt^ and PfERC-*M9*/PMX^apt^ parasites 44 and 48 h after addition of GlcN as described for panel C. Data were normalized to the ratio of processed PMX (p45/p67) of PfERC-*M9*/PMX^apt^ parasites and are presented as means ± standard errors of the means (*n* = 6 [44 h] and *n* = 11 [48 h] biological replicates; ***, *P* < 0.001; ****, *P* < 0.0001 [2-way ANOVA]). (E) A proposed model for the role of PfERC in egress. PfERC regulates the activity an unknown protease (Protease Y) that functions upstream of PMX. PMX then cleaves SUB1, eventually leading to egress of the parasite. Knockdown of PfERC inhibits the activity of protease Y, thus inhibiting the *Plasmodium* egress proteolytic cascade.

10.1128/mBio.03078-19.6 FIG S6 Representative images of Western blots of lysates from PfERC-*glmS* and PfERC-*M9* schizonts incubated with GlcN for 48 h and probed with anti-SUB1, anti-MSP1 12.4 and 9.2, anti-AMA1, anti-RAP1, and anti-V5 antibodies from the experiments whose results are presented in Fig. 4 to 7. The sizes of the marker proteins that comigrated with the probed protein are indicated on the left. Download FIG S6, PDF file, 2.0 MB. Copyright © 2020 Fierro et al. 2020 Fierro et al. This content is distributed under the terms of the Creative Commons Attribution 4.0 International license.

Even though we did not observe any defects in protein localization upon PfERC knockdown, observation of organelle morphology was not possible in these immunofluorescence assays (IFAs). As the ER produces the lipid membranes required for generating organelles essential for egress and invasion, we observed organelle morphology after knockdown using TEM. As described above, knockdown was initiated in synchronized PfERC-*glmS* and PfERC-*M9* schizonts, and after 48 h, these schizonts were treated with C1 for 4 h. Then, we observed these C1-treated schizonts using TEM (Fig. 4G; see also Fig. S3C). The micronemes and rhoptries were morphologically identical in these PfERC-*glmS* and PfERC-*M9* schizonts, demonstrating that PfERC knockdown did not cause a defect in organelle biogenesis (Fig. 4G; see also Fig. S3C).

### PfERC is required for AMA1 processing but not for secretion.

Studies have shown that secretion of micronemes require Ca^2+^ signaling pathways (9), and, although our data suggested that PfERC is not required for Ca^2+^ storage in the ER, we could not rule out a role for PfERC in Ca^2+^ signaling. The invasion ligand apical membrane antigen 1 (AMA1) localizes to micronemes and is translocated to the merozoite membrane via exocytosis of micronemes upon encountering a Ca^2+^ signal (6, 12). Since the translocation of AMA1 requires the putative egress Ca^2+^ signaling pathway, we observed whether AMA1 exocytosis was inhibited upon PfERC knockdown (6, 12). Synchronized PfERC-*M9* or PfERC-*glmS* schizonts for which knockdown had been initiated during the previous cycle were incubated with C1 to achieve tight synchronization. Then, C1 was washed off and the parasites were incubated with E-64 to trap merozoites that had initiated egress within the RBC membrane (12). Using SIM, we observed AMA1 localization either in micronemes or on the surface of merozoites (Fig. 5A). Our data show that there were no differences between the PfERC-*M9* and PfERC-*glmS* parasites in the localization of AMA1, suggesting that PfERC does not function in the signaling pathway required for vesicle secretion (Fig. 5A and B).

The proteolytic processing of AMA1 is required for the formation of the tight junction between the parasite and the RBC (35–37). After AMA1 is trafficked from micronemes to the merozoite surface prior to egress, it is processed from its proform (p83) to its mature form (p66) by an unknown protease (38–40). Therefore, we tested if the processing of the AMA1 was inhibited upon knockdown of PfERC. After initiating knockdown in synchronized schizonts, lysates from second-cycle PfERC-*glmS* and PfERC-*M9* schizonts were separated on a Western blot and probed with anti-AMA1 antibodies (Fig. 5C; see also Fig. S6B). We observed a significant (>40%) reduction in the proteolytic processing of AMA1 upon PfERC knockdown in PfERC-*glmS* mutants compared to the PfERC-*M9* control (Fig. 5C and D). These data suggest that PfERC is required for the proteolytic maturation of AMA1 during egress.

### SUB1 maturation requires PfERC.

Though the protease responsible for the maturation of AMA1 is unknown, it occurs downstream of the egress proteolytic cascade (16, 17). Further, our electron microscopy data show that knockdown of PfERC prevents the breakdown of the PVM (Fig. 3). Therefore, we observed the proteolytic processing of SUB1, which is required to start a proteolytic cascade that breaks down the PVM and RBCM during egress (14, 41). SUB1 is processed twice. The first processing step takes place in the ER, where it undergoes Ca^2+^-dependent autocatalytic processing from its zymogen (83-kDa) form into a 54-kDa semiproenzyme form (p54) (18, 19, 42). From the ER, SUB1 is transported to egress-related secretory vesicles called exonemes, which are secreted into the PV to initiate breakdown of the PVM. It has been suggested that during trafficking of SUB1 to exonemes (or in the exoneme itself), SUB1 is processed by PMX from its semiproenzyme form (p54) to its mature form (p47) (16, 17). The secretion of the mature p47 form of SUB1 then initiates the breakdown of the PVM (14, 42). Given that one CREC family member has been shown to transiently interact with a subtilisin like protease in mammalian cells (25), we hypothesized that PfERC is required for one of the proteolytic maturation steps of SUB1, most likely the first Ca^2+^-dependent autocatalytic processing step in the ER.

To test this hypothesis, PfERC-*glmS* and PfERC-*M9* schizonts were incubated with GlcN and allowed to progress through one asexual growth cycle (48 h) to develop into schizonts again. Lysates from these synchronized schizonts were separated on a Western blot and probed with antibodies against SUB1 (Fig. 6A; see also Fig. S6C). No change was observed in the Ca^2+^-dependent autoprocessing from the zymogen form of SUB1 into the semiproenzyme (p54) form (Fig. S6C). Surprisingly, we observed a reproducible and significant decrease in the level of processing of SUB1 from the p54 form to the p47 form in PfERC-*glmS* parasites (Fig. 6A and B). Compared to PfERC-*M9* parasites, there was a >50% decrease in the amount of processed SUB1 (p47) in PfERC-*glmS* parasites (Fig. 6B). This effect was also observed in cells treated with compound 1, suggesting that PfERC is required for SUB1 processing prior to secretion of exonemes (Fig. 6C and D). Taken together, our data suggest that PfERC is essential for the proteolytic maturation of SUB1.

Since we observed the presence of some mature SUB1 in PfERC-*glmS* parasites (Fig. 6A), we tested if the activity of SUB1 was inhibited upon knockdown of PfERC by assaying for the processing of a known SUB1 substrate, MSP1. MSP1 is a GPI-anchored merozoite membrane protein that is presumably processed by SUB1 once the protease is secreted into the PV (32, 41). It is required for the initial attachment of merozoites onto RBCs, and it was shown previously that correct processing of MSP1 by SUB1 is also required for efficient egress as it plays a role in breakdown of the RBC cytoskeleton (32, 43–45). Lysates from synchronized second-cycle PfERC-*glmS* and PfERC-*M9* schizonts, treated as described above, were separated on a Western blot and probed using anti-MSP1 antibodies (Fig. 6E; see also Fig. S6D and E). Our data show that there was significant inhibition of MSP1 processing in PfERC-*glmS* parasites compared to PfERC-*M9* parasites after knockdown (Fig. 6F; see also Fig. S6D and E). These data reveal that knockdown of PfERC leads to defects in SUB1 processing and activity and, consequently, in MSP1 processing.

Together, these data suggest that the knockdown of PfERC specifically inhibited the proteolytic maturation of SUB1. Since SUB1 binds Ca^2+^ and since this is required for its activity, one possibility is that PfERC directly regulates the proteolysis of SUB1. In mammalian cells, CREC family members have been shown to interact transiently with a subtilisin-like protease (25). Therefore, to test if PfERC interacts directly with SUB1, we performed coimmunoprecipitation (co-IP) experiments but failed to detect any interaction between these two proteins (Fig. S7A).

10.1128/mBio.03078-19.7 FIG S7 Coimmunoprecipitation of PfERC and SUB1 or PMX. PfERC-*M9* (A) and PfERC-*M9*/PMX^apt^ (B) schizonts were isolated, and PfERC was immunoprecipitated using HA-conjugated beads. The input, IP, and unbound fractions were probed with anti-HA and anti-SUB1 or anti-V5 (PMX) antibodies. (B) An artifact from an HA antibody heavy chain was observed in the IP sample. Download FIG S7, PDF file, 0.7 MB. Copyright © 2020 Fierro et al. 2020 Fierro et al. This content is distributed under the terms of the Creative Commons Attribution 4.0 International license.

### PfERC knockdown inhibits PMX maturation.

It is possible that the interaction between PfERC and SUB1 is too transient to detect using co-IP (Fig. S7A). Another possibility is that PfERC works upstream of SUB1 in the egress proteolytic cascade via the recently discovered aspartic protease PMX (16, 17). To test this, we generated double conditional mutant parasites where we tagged PMX with a C-terminal V5 tag and regulated its expression using *tetR* aptamers in both PfERC-*glmS* and PfERC-*M9* parasites (termed PfERC-*glmS*/PMX^apt^ and PfERC-*M9*/PMX^apt^) (Fig. 7A) (46). This aspartic protease undergoes proteolytic maturation from a 67-kDa zymogen to a 45-kDa active protease (16, 17). Our data show that we were successful in tagging PMX with a V5 tag in PfERC-*glmS*/PMX^apt^ and PfERC-*M9*/PMX^apt^ parasites (Fig. 7B; see also Fig. S8).

10.1128/mBio.03078-19.8 FIG S8 Validation of PfERC/PMX double mutants. (A) Results of PCR analysis of the generated mutants (2 clones each from the PfERC-*glmS* background and the PfERC-*M9* background) using specific primers (P14+P11; Table S1) in the C-terminal and aptamer regions show integration of the plasmid into the PMX locus. Results of PCR analysis performed using specific primers in the C terminus and the 3′UTR of PMX show the absence of wild-type parasites in the clonal population; results of PCR analysis specific to the aptamer region (P15+P16) show the correct number of aptamers in our clones. (B) Representative Western blotting of lysates isolated from asynchronous parental PfERC-*glmS* and PfERC-*M9* clones as well as the PfERC-*glmS*/PMX^apt^ and PfERC-*M9*/PMX^apt^ double mutants probed with anti-V5 antibodies shows that the PMX gene was tagged with V5 in the double mutants but not in the parental line. Download FIG S8, PDF file, 0.4 MB. Copyright © 2020 Fierro et al. 2020 Fierro et al. This content is distributed under the terms of the Creative Commons Attribution 4.0 International license.

Since our data suggested that PfERC works upstream of SUB1 and that PMX functions upstream of SUB1 in the egress cascade, we tested whether PfERC knockdown inhibited PMX trafficking or its proteolytic maturation or both. After initiating PfERC knockdown in synchronized PfERC-*glmS*/PMX^apt^ and PfERC-*M9*/PMX^apt^ schizonts, we observed the subcellular localization of PMX in the second-cycle schizonts (Fig. S9). Immunofluorescence microscopy revealed that trafficking of PMX to exonemes was not defective in our mutants upon PfERC knockdown. Next, we observed the proteolytic maturation of PMX from a 67-kDa zymogen to a 45-kDa active protease in our double mutants (16, 17). As described above, after initiation of PfERC knockdown in synchronized schizonts, we isolated lysates from second-cycle PfERC-*glmS*/PMX^apt^ and PfERC-*M9*/PMX^apt^ schizonts and monitored PMX cleavage by Western blotting. We observed a reproducible and significant decrease in the processing of PMX from p67 to the p45 form in PfERC-*glmS*/PMX^apt^ parasites at both 44 and 48 h after addition of GlcN (Fig. 7C and D; see also Fig. S6F). No such deficiencies in PMX cleavage were observed in PfERC-*M9*/PMX^apt^ parasites (Fig. 7C and D). Together, these data confirm a specific defect in PMX processing but not a defect in its trafficking.

10.1128/mBio.03078-19.9 FIG S9 Trafficking of PMX in PfERC/PMX double mutants. Representative SIM images of PfERC-*glmS*/PMX^apt^ and PfERC-*M9*/PMX^apt^ double mutants grown in the presence of GlcN for 48 h and then stained with anti-V5 antibodies as well as the nuclear stain (*n* = 2 biological replicates) are shown. From top to bottom, the images represent DAPI (blue), anti-V5 (green), and fluorescence merge. Bar, 2 μm. Download FIG S9, PDF file, 2.4 MB. Copyright © 2020 Fierro et al. 2020 Fierro et al. This content is distributed under the terms of the Creative Commons Attribution 4.0 International license.

The processing defect of PMX might be explainable by a direct interaction between PfERC and PMX. Therefore, to test this hypothesis, we performed co-IP assays using anti-HA antibodies to immunoprecipitate PfERC (Fig. S7B). However, we did not observe an interaction between PMX and PfERC, suggesting that there are as-yet-undiscovered proteins in the egress proteolytic cascade (Fig. S7B). These data are consistent with a model whereby PfERC controls egress by interacting with a protease upstream of PMX. Removal PfERC interfered with the function of this unknown protease required for PMX maturation and thus inhibited egress (Fig. 7E). Together, these data show that PfERC is essential for the proteolytic maturation of PMX during egress, placing it as the earliest known regulator in the egress proteolytic cascade in malaria parasites.

## DISCUSSION

In this study, we discovered the biological function of a conserved Ca^2+^-binding protein that resides in the lumen of the ER of Plasmodium falciparum. Our data show that PfERC is essential for asexual replication of malaria parasites. Knockdown of PfERC did not affect the ring and trophozoite development but clearly inhibited the subsequent schizont-to-ring transition. Specifically, these data show that PfERC is required for egress from infected RBCs and potentially for invasion into host erythrocytes. This is consistent with data that suggest PfERC may be transcriptionally controlled by the invasion-specific transcription factor PfAP2-I (47). Knockdown of PfERC leads to defects in the processing of proteins critical for invasion of merozoites into the host RBC, namely, MSP1 and AMA1. However, any invasion defect is likely a secondary effect because several proteins critical for invasion are processed during egress (16, 17, 32, 41). Given the kinetic limitations of the conditional knockdown system, we cannot tease out a specific role for PfERC in invasion. As invasion occurs rapidly (<2 min), a potential specific invasion-related function of PfERC can be tested using a small molecule that specifically targets PfERC (48). Overall, these data show that PfERC is essential for egress of merozoites from the infected RBC.

As an ER-resident protein, knockdown of PfERC could affect several ER functions such as protein trafficking, organellar biogenesis, and Ca^2+^ signaling. Therefore, we tested if PfERC functions in the trafficking of proteins that localize to different subcellular organelles and that are required for the schizont-to-ring transition such as MSP1, AMA1, EBA175, PMX, and RAP1. A defect in the secretory pathway would explain the observed deficiencies in the proteolytic processing of PMX, SUB1, MSP1, and AMA1, as transport from the ER is required for their maturation (6, 19, 34). However, SIM and electron microscopy experiments show that proteins on the merozoite surface, micronemes, and rhoptries are trafficked normally and that the biogenesis of egress and invasion organelles is normal. Likewise, Western blotting showed that the proteolytic processing of a rhoptry protein, RAP1, which is processed by PMIX after transport to the organelle, occurs normally upon knockdown of PfERC (16, 17). Further, this suggests that PfERC knockdown did not inhibit the function of PMIX. These data show that knockdown of PfERC does not result in a generalized defect in protein trafficking via the ER or in organelle biogenesis.

CREC family members in the ER are known to regulate the function of Ca^2+^ pumps and channels such as the ryanodine and IP_3_ receptors (49, 50). Therefore, one interesting possibility that we considered was that PfERC may play a role in the signal-dependent release of Ca^2+^ from the ER. This is difficult to test in *Plasmodium* since there are no clear orthologs for a ligand-dependent Ca^2+^ channel in its genome (51). Intracellular Ca^2+^ stores are required for egress and invasion of malaria parasites since cell-permeable Ca^2+^ chelators block egress of *Plasmodium* parasites from host RBCs (13, 52–55). Further, Ca^2+^ binding proteins in the parasite cytoplasm are essential for egress of malaria parasites. For example, the Ca^2+^-dependent protein kinase PfCDPK5 is required for secretion of egress-specific organelles, such as those containing AMA1 (12, 56). As PfCDPK5 is thought to be activated upon the signal-dependent release of intracellular Ca^2+^ into the cytoplasm (12), we tested if PfERC was required for exocytosis of AMA1-containing vesicles. The data suggest that PfERC is not required for the PfCDPK5-dependent translocation of AMA1 onto merozoite membrane. However, PfERC is required for the essential proteolytic maturation of AMA1, suggesting that this CREC family member regulates (directly or indirectly) the unknown protease that cleaves AMA1.

An essential enzyme vital for initiating egress is the protease SUB1, as this serine protease is required for the rupture of both the PVM and the RBCM (14, 41). SUB1 is produced as an 82-kDa zymogen in the ER, where it rapidly self-processes into a 54-kDa semiproenzyme in the ER (42). If PfERC was needed for this autoprocessing event, then this would explain the observed knockdown phenotypes (41). Instead, our data show that PfERC was essential for the second processing step of SUB1, which produces the mature, active form of the protease (p54 to p47). This processing event occurs once trafficking from the ER has occurred, suggesting a role for PfERC in SUB1 maturation once it leaves the ER (6, 13, 17, 18). The release of the mature SUB1 into the PV kick-starts the egress cascade (41). The exocytosis of SUB1-containing vesicles is thought to occur upon the activation of the cGMP signaling pathway and the release of intracellular Ca^2+^ stores (54). Therefore, we tested whether PfERC plays a role in the cGMP signaling pathway, using the PKG inhibitor compound 1, and show that PfERC knockdown inhibited SUB1 maturation even when PKG activity was inhibited. These data, together with the experiments testing AMA1 translocation onto the merozoite membrane, suggest that PfERC does not function in the signal-dependent exocytosis of egress-specific organelles. Instead, our data suggest a model where PfERC plays a specific role in the maturation of SUB1 prior to its secretion into the PV.

One possibility is that PfERC is required for the maturation of a protease in this pathway that works upstream of SUB1. The recently discovered aspartic protease PMX cleaves SUB1 from p54 to p47 (16, 17). In turn, PMX itself is proteolytically processed in several distinct steps from a 67-kDa zymogen to a 45-kDa active protease (16, 17). But unlike most aspartyl proteases, PMX does not appear to perform autoprocessing because inhibitors that block PMX activity do not inhibit its maturation (16). However, since PMX processing occurs in several steps, it is possible that there may be one or more proteases involved in this process. Alternatively, PMX maturation may require a combination of autoprocessing and the involvement of other proteases, as is the case for SUB1. The protease responsible for PMX maturation is unknown, and there are no obvious candidates among the secreted proteases in the *Plasmodium* genome. Our data show that PfERC knockdown inhibits the maturation of PMX, suggesting that PfERC functions upstream in the egress proteolytic cascade to regulate the protease required for PMX cleavage. We propose that PfERC acts as Ca^2+^-dependent switch to bind and regulate the activity of a protease upstream of PMX. Thus, in the absence of PfERC, the egress proteolytic cascade is inactive, thus inhibiting the proteolytic cleavage of PMX and SUB1. Since our data suggest that PfERC does not play a role in signal-dependent vesicle exocytosis, it is likely that immature PMX and SUB1 are released from exonemes into the PV. However, since the unprocessed forms of PMX and SUB1 are inactive, they fail to break down the PVM as well as to cleave essential invasion ligands such as MSP1 and AMA1 on the merozoite surface.

A principal finding of these studies was the discovery of an early regulator in the ER of P. falciparum with a specific role in egress of malaria parasites from RBCs and potentially in the invasion of parasites into the RBC. These data help build a model where PfERC modulates the maturation of the egress proteolytic cascade. These studies lay the foundation for understanding the vital and key role that ER-resident proteins play in the egress of human malaria parasites from the infected RBC and in their reentry into the host cell. Some studies have suggested that a key class of antimalarials containing endoperoxides, including the frontline antimalarial artemisinin, may target PfERC (48) and that one of the transcriptomic responses of artemisinin-resistant parasites is the overexpression of PfERC (57). These data suggest that targeting PfERC (and thus egress) is a viable strategy for antimalarial drug development.

## MATERIALS AND METHODS

### Cell culture and transfections.

*Plasmodium* parasites were cultured in RPMI 1640 medium supplemented with AlbuMAX I (Gibco) and transfected as described earlier (58–61). To generate PfERC*-glmS* and PfERC*-M9* parasites, a mix of two plasmids (50 μg each) was transfected in duplicate into 3D7 parasites. The plasmid mix contained plasmid pUF1-Cas9-guide (62), which contains the DHOD resistance gene, and plasmid pPfERC-HA-SDEL-*glmS* or pPfERC-HA-SDEL-*M9*, each of which is marker-free. Drug pressure was applied 48 h after transfection, using 1 μM DSM1 (63) and selecting for Cas9 expression. DSM1 was removed from the culturing medium once the parasites were detected in the culture, around 3 weeks posttransfection.

To generate PfERC-*glmS*/PMX^apt^ and PfERC-*M9*/PMX^apt^ parasites, we transfected PfERC-*glmS* and PfERC-*M9* parasites with a mix of two plasmids: plasmid pPMX-V5-Apt-pMG74 and the pyAIO plasmid containing the PMX guide RNA (16). Before transfection, 20 μg of pPMX-V5-Apt-pMG74 was digested overnight with EcoRV. The enzyme was then subjected to heat inactivation for 20 min at 65°C and DNA was coprecipitated with 50 μg of PMX guide RNA pyAIO. Transfected parasites were grown in 0.5 μM anhydrous tetracyline (aTc) (Cayman Chemical). Drug pressure was applied 48 h after transfection, using BSD at a concentration of 2.5 μg/ml (63), selecting for pPMX-V5-Apt-pMG74 expression. Two clones each were isolated from the PfERC-*glmS* and the PfERC-*M9* backgrounds.

### Construction of PfERC and PMX plasmids.

Genomic DNA was isolated from P. falciparum cultures by the use of a QIAamp DNA blood kit (Qiagen). Constructs utilized in this study were confirmed by sequencing. PCR products were inserted into the respective plasmids using an In-Fusion cloning system (Clontech), the sequence- and ligation-independent cloning (SLIC) method (60, 61), T4 ligation (New England BioLabs), or site-directed mutagenesis by the use of QuikChange (Agilent). To generate the pHA-SDEL-*glmS*/*M9* plasmid, primers 1 and 2 were used to add an ER retention signal (SDEL) at the end of the HA tag in pHA-*glmS* and pHA-*M9* plasmids (60, 61).

For generating the PfERC-*glmS*/*M9* conditional mutants, pHA-SDEL-*glmS*/*M9* plasmid, consisting of two homology regions flanking the HA-SDEL tag and the *glmS* or *M9* sequence, was used as a donor DNA template. To allow efficient genomic integration of the pHA-SDEL-*glmS* and pHA-SDEL-*M9* donor plasmids, 800-bp sequences were used for each homology region. The C terminus of the *pferc* coding region was PCR amplified from genomic DNA using primers 3 and 4 (containing the shield mutation) and was inserted into pHA-SDEL-*glmS* and pHA-SDEL-*M9* using restriction sites SacII and AfeI. The 3′ untranslated region (3′UTR) of *pferc* was PCR amplified from genomic DNA using primers 5 and 6 and was inserted into pHA-SDEL-*glmS* and pHA-SDEL-*M9* (already containing the C-terminal region) by the use of restriction sites HindIII and NheI. For expression of PfERC guide RNA, oligonucleotides 7 and 8 were inserted into plasmid pUF1-Cas9-guide as previously described (60, 61). Briefly, plasmid pUF1-Cas9-guide was digested with BtgZI and annealed oligonucleotides were inserted using SLIC. Primers 3 and 6 and primers 3 and 9 (which recognize the *glmS*/*M9* sequence) were used for clone verification.

To generate the pPMX-V5-Apt-pMG74 plasmid, primers 10 and 11 were used to amplify the 3′UTR homology region containing AflII and EcoRV sites, respectively, and primers 12 and 13 were used to amplify the C-terminal homology region containing EcoRV and PspXI sites, respectively. Then, using primers 10 and 13, the two PCR products were stitched together by PCR. This final PCR product was ligated into plasmid pPMX-V5-Apt-pMG74 by the use of the AflII and PsPXI sites and SLIC.

### *Plasmodium* growth assays.

Asynchronous growth assays were done as described previously (60, 61). Briefly, 5 mM glucosamine (GlcN) (Sigma) was added to the growth medium and parasitemia was monitored every 24 h using a CyAn ADP (Beckman Coulter) or CytoFLEX (Beckman Coulter) flow cytometer and analyzed by FlowJo software (Tree Star, Inc.). Data were fit to exponential-growth equations using Prism (GraphPad Software, Inc.).

To determine the ring/schizont ratio of PfERC-*glmS* and PfERC-*M9* parasites, 7.5 mM GlcN was added to Percoll gradient-isolated schizont-stage parasites, and the parasites were allowed to egress and reinvade fresh RBCs. Two hours later, 5% sorbitol–7.5 mM GlcN was added to the invaded culture to lyse any remaining schizonts and isolate 2-h rings. The ring-stage parasites were grown again in media supplemented with GlcN. Samples were then taken at 44 h, 48 h, and 56 h and read by flow cytometry to determine the population of rings and schizonts present at those times using FlowJo software (Tree Star, Inc.). To determine the development of each intraerythrocytic stage during the asexual life cycle of PfERC-*glmS* and PfERC-*M9* parasites, 7.5 mM was added to Percoll gradient-isolated schizont-stage parasites, and the parasites were allowed to egress and reinvade fresh RBCs. At specific times, Hema-3-stained blood smears were used to count parasite stages and the percentage of the specific life cycle stage was calculated as follows: %of stages=number of specific stagestotal number of parasites. The time when GlcN was added was designated 0 h.

To determine the amount (percentage) of rings or schizonts, samples of synchronized schizonts grown with 7.5 mM GlcN for about 48 h were taken and fixed with 8% paraformaldehyde and 0.3% glutaraldehyde. Samples were read by flow cytometry. For growth assays using compound 1, synchronized schizonts were grown with 7.5 mM GlcN for about 48 h. Then, schizonts were subjected to Percoll gradient isolation and incubated with compound 1 for 4 h and then removed by gentle washing of the parasites twice with 1 ml of warm, complete RPMI 1640 medium–7.5 mM GlcN. Parasites were resuspended with fresh media and RBCs, and fixed samples (processed as described above) were read by flow cytometry. DNA content was determined using Hoechst 33342 staining (Thermo Fisher).

### Western blotting.

Western blotting was performed for *Plasmodium* parasites as described previously (60, 61). Briefly, parasites were permeabilized selectively by treatment with ice-cold 0.04% saponin–phosphate-buffered saline (PBS) for 10 min and pellets were collected for detection of proteins with the parasite. For detection of MSP1, schizonts were isolated on a Percoll gradient (Genesee Scientific) and whole-cell lysates were generated by sonication with radioimmunoprecipitation (RIPA) buffer (150 mM NaCl, 20 mM Tris-HCl [pH 7.5], 1 mM EDTA, 1% SDS, 0.1% Triton X-100). The antibodies used in this study were rat anti-HA (3F10; Roche) (1:3,000), rabbit anti-HA (715500; Invitrogen) (1:100), mouse anti-V5 (TCM5; eBioscience) (1:1,000), rabbit anti-PfEF1α (from D. Goldberg) (1:2,000), mouse anti-plasmepsin V (from D. Goldberg) (1:400), rabbit anti-SUB1 (from Z. Dou and M. Blackman) (1:10,000), rat anti-AMA1 (28G2; Alan Thomas via BEI Resources, NIAID, NIH) (1:500), mouse anti-MSP1 (12.4; European Malaria Reagent Repository) (1:500), and mouse anti-RAP1 (2.29; European Malaria Reagent Repository) (1:500). The secondary antibodies that were used were IRDye 680CW goat anti-rabbit IgG and IRDye 800CW goat anti-mouse IgG (Li-COR Biosciences) (1:20,000). The Western blotting images were processed using Odyssey Clx Li-COR infrared imaging system software (Li-COR Biosciences). Calculation of knockdown and processing ratios was performed by the use of both the Odyssey infrared imaging system software and ImageJ 1.8 (NIH).

### Immunofluorescence microscopy.

For IFAs, cells were fixed as described previously (60, 61). The antibodies used for IFA were as follows: rat anti-HA antibody (clone 3F10; Roche) (1:100), mouse anti-AMA1 (1F9 from Alan Cowman), rat anti-PfGRP78 (MRA-1247; BEI Resources, NIAID, NIH) (1:100), mouse anti-MSP1 (12.4; European Malaria Reagent Repository) (1:500), rat anti-AMA1 (28G2; Alan Thomas via BEI Resources, NIAID, NIH) (1:500), mouse anti-EBA175 (R218; B. Kim Lee Sim via BEI Resources, NIAID, NIH) (1:500), and mouse anti-RAP1 (2.29; European Malaria Reagent Repository) (1:500). Secondary antibodies used were anti-rat antibody conjugated to Alexa Fluor 488 or Alexa Fluor 546 and anti-rabbit antibody conjugated to Alexa Fluor 488 (Life Technologies) (1:100). Cells were mounted on ProLong diamond with 4′,6-diamidino-2-phenylindole (DAPI) (Invitrogen) and imaged using a Delta-Vision II microscope system with an Olympus IX-71 inverted microscope using a 100× objective or an Elyra S1 SR-SIM microscope (Zeiss). Image processing, analysis, and display were performed using SoftWorx or Axiovision and Adobe Photoshop. Adjustments to brightness and contrast were made for display purposes.

### AMA1 translocation assays.

To observe AMA1 translocation in our mutants, 7.5 mM GlcN was added to Percoll gradient-isolated schizont-stage parasites and parasites were allowed to egress and reinvade fresh RBCs. At 44 to 48 h later, schizonts were subjected to Percoll gradient purification and incubated with 1.5 μM compound 1 for 4 h at 37°C. Then, compound 1 was removed by washing the parasites twice with 1 ml of warm complete RMPI 1640 medium–7.5 mM GlcN. These parasites were immediately resuspended in media plus 7.5 mM GlcN and 20 μM E-64 (Sigma) and incubated at 37°C in a still incubator for 6 h. Parasites were then fixed as described previously (60, 61) and probed with anti-AMA1 (1F9) antibodies. Images were taken using a Delta-Vision II microscope system with an Olympus IX-71 inverted microscope using a 100× objective and an Elyra S1 SR-SIM microscope (Zeiss).

### Immunoprecipitation assay.

For IP, we took samples of Percoll gradient-purified schizonts form PfERC-*M9* parasites. The collected schizonts were incubated on ice for 15 min in extraction buffer (40 mM Tris-HCl, 150 mM KCl, 1 M EDTA plus 1× HALT and 0.5% NP-40). The parasites were lysed via sonication, and the sample was centrifuged at 20,000 × *g* in 4°C for 15 min. The supernatant was mixed with anti-HA antibody-conjugated magnetic beads (Thermo Scientific) for 1 h at room temperature. The beads were washed 3× with IgG binding buffer (20 mM Tris-HCl, 150 mM KCl, 1 mM EDTA, 0.1% NP-40), and elution was performed with 100 μl of 2 mg/ml HA peptide (Thermo Scientific) with rocking at 37°C for 10 min.

### Transmission electron microscopy.

GlcN (7.5 mM) was added to Percoll gradient-isolated schizont-stage parasites, and the parasites were allowed to egress and reinvade fresh RBCs. After 48 h, parasites were subjected to Percoll gradient isolation and then incubated with 2 μM compound 1 for 4 h without shaking at 37°C in an incubator. After incubation, parasites were washed twice with warm, complete RPMI 1640 medium–7.5 mM GlcN.

Samples were taken immediately after washing off compound 1 and then 30 min after. For fixation, parasites were washed with 1× PBS and gently resuspended in 2.5% glutaraldehyde–0.1 M sodium cacodylate–HCl (Sigma) buffer (pH 7.2) for 1 h at room temperature. Parasites were then rinsed in 0.1 M cacodylate-HCl buffer before agar-enrobing the cells in 3% Noble agar. Parasites were postfixed in 1% osmium tetroxide–0.1 M cacodylate–HCl buffer for 1 h and rinsed in buffer and deionized water. Dehydration of the parasite samples was done with an ethanol series, and the samples were then exposed to propylene oxide before infiltration was performed with Epon-Araldite. The blocks of parasites were trimmed, and sections were obtained using a Reichert Ultracut S ultramicrotome (Leica, Inc., Deerfield, IL). Sections (60 to 70 nm thick) were placed on 200-mesh copper grids and poststained with ethanolic uranyl acetate and Reynolds lead citrate. Grids were viewed with a JEOL JEM-1011 transmission electron microscope (JEOL USA, Inc., Peabody, MA) using an acceleration voltage of 80 KeV. Images were acquired using an AMT XR80M wide-angle multidiscipline midmount charge-coupled-device (CCD) camera (Advanced Microscopy Techniques, Woburn, MA).

### Scanning electron microscopy.

GlcN (7.5 mM) was added to Percoll gradient-isolated schizont-stage parasites, and the parasites were allowed to egress and reinvade fresh RBCs. At 48 h later, the parasites were subjected to Percoll gradient isolation and then incubated with 2 μM compound 2 {4-[7- [(dimethylamino)methyl]-2-(4-fluorphenyl)imidazo[1,2-a]pyridine-3-yl]pyrimidin-2-amine} for 4 h without shaking at 37°C in an incubator. After incubation, parasites were washed twice with warm, complete RPMI 1640 medium–7.5 mM GlcN. Samples were taken immediately after washing off compound 2 and then 30 min after and were fixed as described for the TEM samples. Parasites were rinsed with 0.1 M cacodylate–HCl buffer and then placed on glass coverslips prepared with 0.1% poly-l-lysine. Parasites were allowed to settle onto the glass coverslips in a moist chamber overnight and then postfixed in 1% osmium tetroxide–0.1 M cacodylate-HCl buffer for 30 min. Cells on coverslips were rinsed three times in deionized water and then dehydrated with an ethanol series. The glass coverslips were subjected to critical point drying in an Autosamdri-814 critical point dryer (Tousimis Research Corporation, Rockville, MD), mounted onto aluminum pin stubs with colloidal paint, and sputter coated with gold-palladium with a Leica EM ACE600 coater (Leica Microsystems Inc., Buffalo Grove, IL). Stubs were examined with an FEI Teneo field emission scanning electron microscope (FE-SEM) (FEI, Inc., Hillsboro, OR) using the secondary electron detector to obtain digital images.

### Calcium measurements.

To measure Ca^2+^ levels in PfERC mutants, knockdown was induced on synchronized schizonts. After 48 h, schizonts were subjected to Percoll purification and permeabilized selectively by treatment with ice-cold 0.04% saponin–PBS for 10 min. Isolated parasites were then washed two times with BAG buffer (116 mM NaCl, 5.4 mM KCl, 0.8 mM MgSO_4_·7H_2_O, 50 mM HEPES, 5.5 mM glucose) plus 7.5 mM GlcN and incubated with 10 μM Fluo-4AM (Thermo Fisher) with rocking for 45 min at 37°C. After incubation, cells were washed two times with BAG buffer plus 7.5 mM GlcN, placed on ice, and immediately taken for fluorimetric measurements. Fluorescence measurements were carried out in a cuvette (Sarstedt) containing parasites suspended in 2.5 ml of BAG buffer and 100 μM EGTA (Sigma). The cuvette was placed in a Hitachi F-4500 or F-7100 fluorescence spectrophotometer, and Fluo-4AM excitation was done at 505 nm, with emission read at 530 nm (64). Drugs and reagents were added via the use of a Hamilton syringe. The final concentration of CPA (Sigma) was 3 μM, and that of ionomycin (Sigma) was 2 μM.
