# Supplementary material for: An Endoplasmic Reticulum CREC Family Protein Regulates the Egress Proteolytic Cascade in Malaria Parasites
Source: mBio. 2020 Feb 25;11(1):e03078-19. doi: 10.1128/mBio.03078-19 (PMC7042697; doi:10.1128/mBio.03078-19)
Supplement: FIG S1 [file mBio.03078-19-sf001.pdf]

```
PfERC        1 ---------MMKINLY-------KLLCFICVIFLL------HKNVVRS------GDNMKYN
Cab45        1 MVWPWVAMASRWGPLIGLAPCCLWLLGAVLLMDASARPANHSSTRERVANREENEILPPD
ERC55        1 ---------MRLGPR-------TAALGLLLLCAA------AAGAGKA------EE-LHY-
RCN1         1 -----MARGGRGRRL-------GLALGLLLALVLAPRVLRAKPTVRK------ERVVRPD
RCN3         1 ---------MMWRPS-------VLLLLLLLRHGAQGKPSPDAGPHGQ------GR-VHQA
Calumenin    1 ---------MDLRQF-------LMCLSLCTAFAL------SKPTEKK------DR-VHHE

PfERC       34 DMKGL---DDLSKLNDDQVKDILG----------LKIDGAKERIEKLFHLID--KNNDKE
Cab45       61 HLNGVKLEMDGHLNRGFHQEVFLG-KDLGGFDEDAEPRRSRRKLMVIFSKVD--VNTDRK
ERC55       31 -PLGER-------RSDYDREALLGVQEDVDEYVKLGHEEQQKRLQAIIKKID--LDSDGF
RCN1        43 SELGERPPEDNQ-SFQYDHEAFLG-KEDSKTFDQLTPDESKERLGKIVDRID--NDGDGF
RCN3        38 APLSDAPHDDAHGNFQYDHEAFLG-REVAKEFDQLTPEESQARLGRIVDRMDRAGDGDGW
Calumenin   32 PQLSDKVHNDAQ-SFDYDHDAFLG-AEEAKTFDQLTPEESKERLGKIVSKID--GDKDGF

PfERC       79 ITEEELNTWSSFLKNEIF---LKQVQAEMGQIDSDKDGFISLNELNDAFAQNLDA-KE--
Cab45      118 ISAKEMQRWIMEKTAEHFQEAMEESKTHFRAVDPDGDGHVSWDEYKVKFLASKGH-SEKE
ERC55       81 LTESELSSWIQMSFKHYA---MQEAKQQFVEYDKNSDDTVTWDEYNIQMYDRVIDFDENT
RCN1        99 VTTEELKTWIKRVQKRYI---FDNVAKVWKDYDRDKDDKISWEEYKQATYGYYLG-NPAE
RCN3        97 VSLAELRAWIAHTQQRHI---RDSVSAAWDTYDTDRDGRVGWEELRNATYGHYAP-GE-E
Calumenin   88 VTVDELKDWIKFAQKRWI---YEDVERQWKGHDLNEDGLVSWEEYKNATYGYVLD-DP--

PfERC      133 ------------VEKHSEGLL-----KRFQIVDKDKDGKLSINEVGLLIDPMKDEELKEL
Cab45      177 VADAIRLNEELKVDEETQEVLENLKDRWYQADSPPADLLLTEEEFLSFLHPEHSRGMLRF
ERC55      138 ALDD--------AEEESFRKLHLKDKKRFEKANQDSGPGLSLEEFIAFEHPEEVDYMTEF
RCN1       155 FHDS--------SDHHTFKKMLPRDERRFKAADLNGDLTATREEFTAFLHPEEFEHMKEI
RCN3       152 FHDV--------EDAETYKKMLARDERRFRVADQDGDSMATREELTAFLHPEEFPHMRDI
Calumenin  142 --DP--------DDGFNYKQMMVRDERRFKMADKDGDLIATKEEFTAFLHPEEYDYMKDI

PfERC      176 EINEILEHHDVNKDGKISLDEF-----KQTRSDESSGVKKD-DEMALDDF-NFFDANKDG
Cab45      237 MVKEIVRDLDQDGDKQLSVPEFISLPVGTVENQQGQDIDDNWVKDRKKEFEELIDSNHDG
ERC55      190 VIQEALEEHDKNGDGFVSLEEFL----GDYRWDPTANEDPEWILVEKDRFVNDYDKDNDG
RCN1       207 VVLETLEDIDKNGDGFVDQDEYI----ADMFSHEENGPEPDWVLSEREQFNEFRDLNKDG
RCN3       204 VIAETLEDLDRNKDGYVQVEEYI----ADLYSAEPGEEEPAWVQTERQQFRDFRDLNKDG
Calumenin  192 VVQETMEDIDKNADGFIDLEEYI----GDMYSHDGNTDEPEWVKTEREQFVEFRDKNRDG

PfERC      229 FIDKEEIIKVYFDPAHESGAINVNEIKENIFEGKKITYDLWNEKALKIAVTSLTDYGDVI
Cab45      297 IVTAEELES-YMDPMNEYNALN---------EAKQMIAVADENQNHHLEPEEVLKYSEF-
ERC55      246 RLDPQELLP-WVVPNNQGIAQE---------EALHLIDEMDLNGDKKLSEEEILENPDL-
RCN1       263 KLDKDEIRH-WILPQDYDHAQA---------EARHLVYESDKNKDEKLTKEEILENWNM-
RCN3       260 HLDGSEVGH-WVLPPAQDQPLV---------EANHLLHESDTDKDGRLSKAEILGNWNM-
Calumenin  248 KMDKEETKD-WILPSDYDHAEA---------EARHLVYESDQNKDGKLTKEEIVDKYDL-

PfERC      289 RYPEDFKLDIGKNVILPTARSRAFEDDDMDADNTEDDKDEADDASQQKSPAIDEL
Cab45      346 -FTGSKLVDYARSV-------------------------------------HEEF
ERC55      295 -FLTSEATDYGRQLH-------------------------------DDYFYHDEL
RCN1       312 -FVGSQATNYGEDLT-------------------------------KN---HDEL
RCN3       309 -FVGSQATNYGEDLT-------------------------------RH---HDEL
Calumenin  297 -FVGSQATDFGEALV-------------------------------R----HDEF
```
