# Supplementary material for: An Endoplasmic Reticulum CREC Family Protein Regulates the Egress Proteolytic Cascade in Malaria Parasites
Source: mBio. 2020 Feb 25;11(1):e03078-19. doi: 10.1128/mBio.03078-19 (PMC7042697; doi:10.1128/mBio.03078-19)
Supplement: TABLE S1 [file mBio.03078-19-st001.docx]

| Primer | Primer Sequence |
| --- | --- |
| 1 | CTGCAGGTCTGGACATTTAAAGTTCATCACTAGCGTAATCTGGAACATCG |
| 2 | CGATGTTCCAGATTACGCTAGTGATGAACTTTAAATGTCCAGACCTGCAG |
| 3 | AATTCGCCCTTTCCGCGGAGAATAGAAAAATTATTTCATTTGATAGATAAAAACAATGAT |
| 4 | TGGGTAACTAGTAGCGCTTAATTCATCAATTGCTGGGGATTTTTGTTGCGATGCATCGTC |
| 5 | ATGATCTTGCCGGCAAGCTTTTTATATAAACATATTTTTTTTTTTTTTAACATAAAGGG |
| 6 | CCTTGAGCTCGCTAGCGACAAATTGGATAGATAATAGGGGGTACAAATATACATAC |
| 7 | AAGTATATAATATTCAATTGCTGGGGATTTTTGTGTTTTAGAGCTAGAA |
| 8 | TTCTAGCTCTAAAACACAAAAATCCCCAGCAATTGAATATTATATACTTA |
| 9 | GCAAGATCATGTGATTTCTCTTTGTTCAAGGAGTCACCCCC |
| 10 | CTTTCCGGGCGCGCCTTAAGCTCTTTATTGAATCCCATATTTTAATATACCC |
| 11 | CCCATCATAAGGGATATCGCTTGTTACACTTATTATACTAGTGTATAACCTATTACATC |
| 12 | AGTGTAACAAGCGATATCCCTTATGATGGGAAGTCAACTTTAATAATTGGTGGGATTAG |
| 13 | TTGGTTTACCCTCGAGGTTTTTACTTTTTGCTCTTGCTACTCCAACCATAGAAGGTCTAC |
| 14 | GTAGGCTTAGGATTTCCAGGAATGTTATCAGCTGGGAATATACC |
| 15 | CTTATGACGTACCTGATTATGCAC |
| 16 | GTAGACCCCATTGTGAGTACATAAATATATTATATAAACTAGACTAGG |
