# Supplementary figures and images for: An Endoplasmic Reticulum CREC Family Protein Regulates the Egress Proteolytic Cascade in Malaria Parasites
Source: mBio. 2020 Feb 25;11(1):e03078-19. doi: 10.1128/mBio.03078-19 (PMC7042697; doi:10.1128/mBio.03078-19)

A

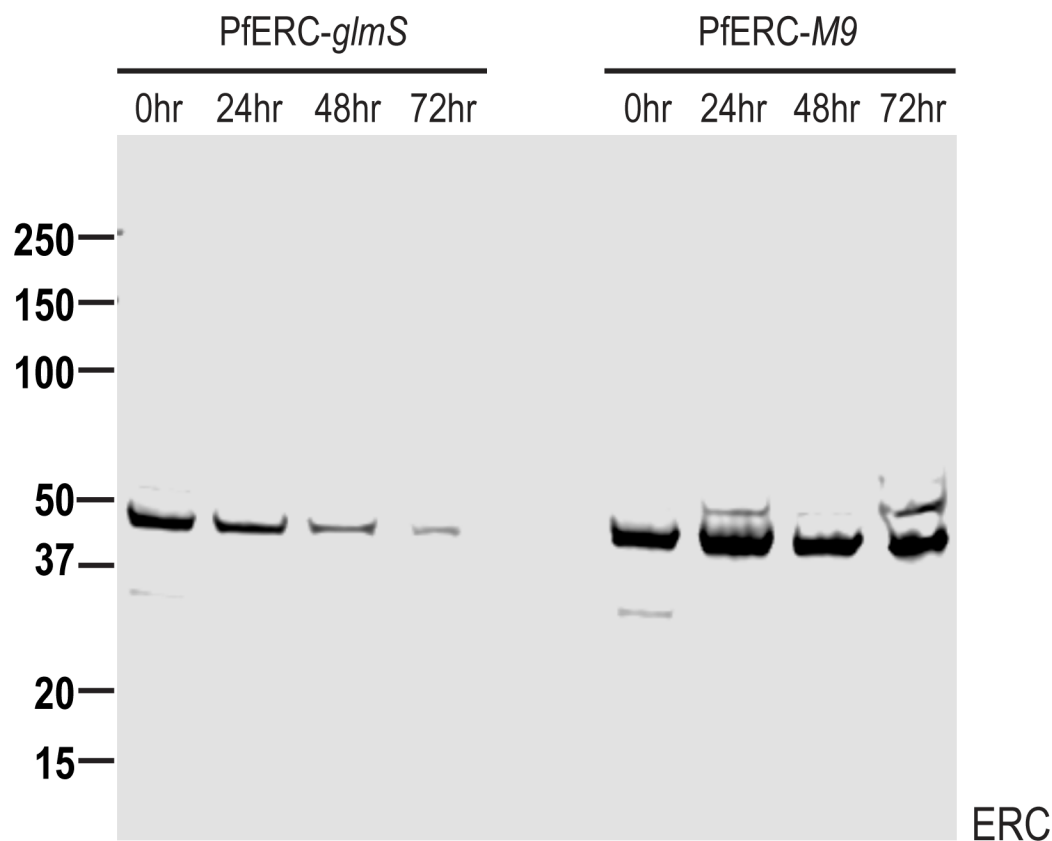

B

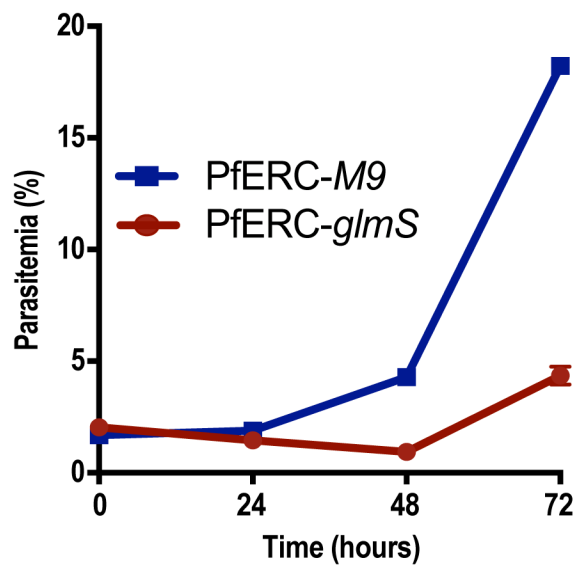

C

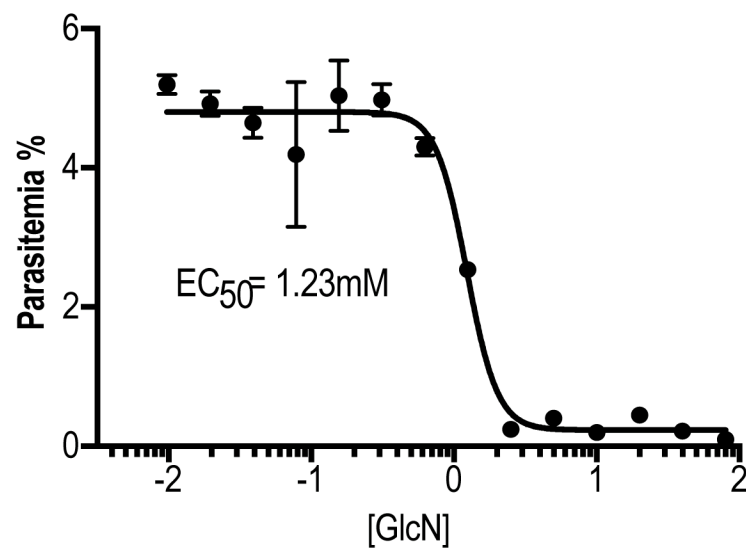

D

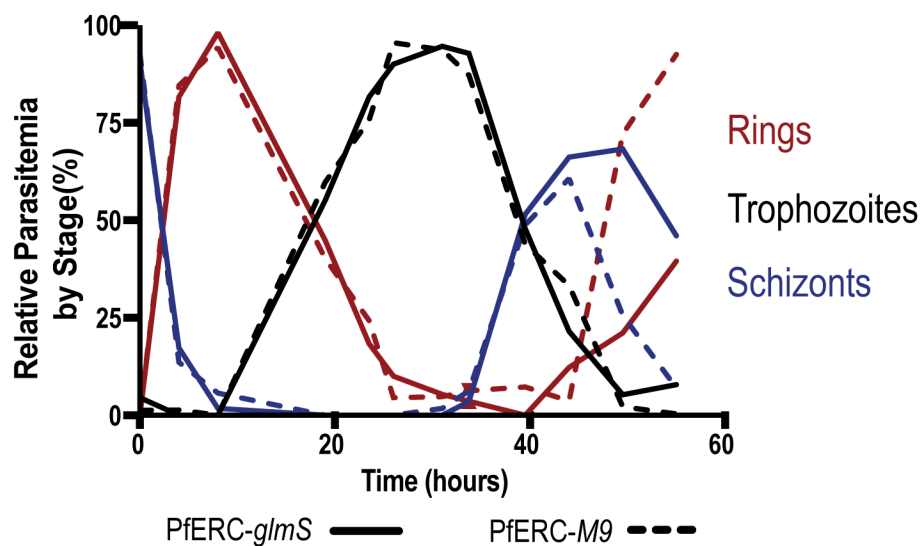

Supplement: FIG S2 [file mBio.03078-19-sf002.pdf]

A

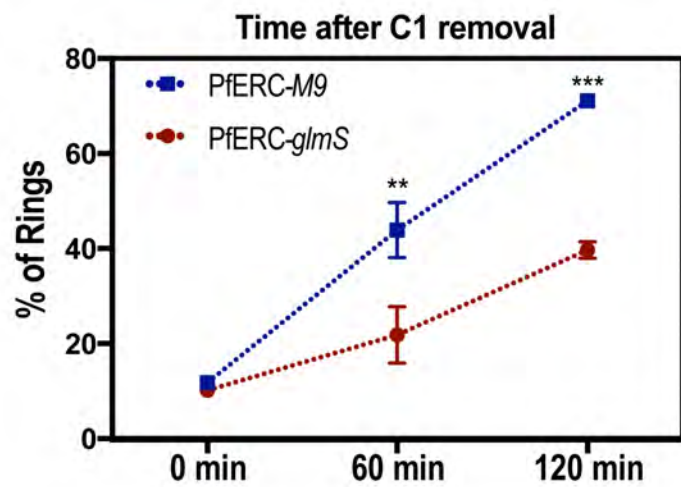

B

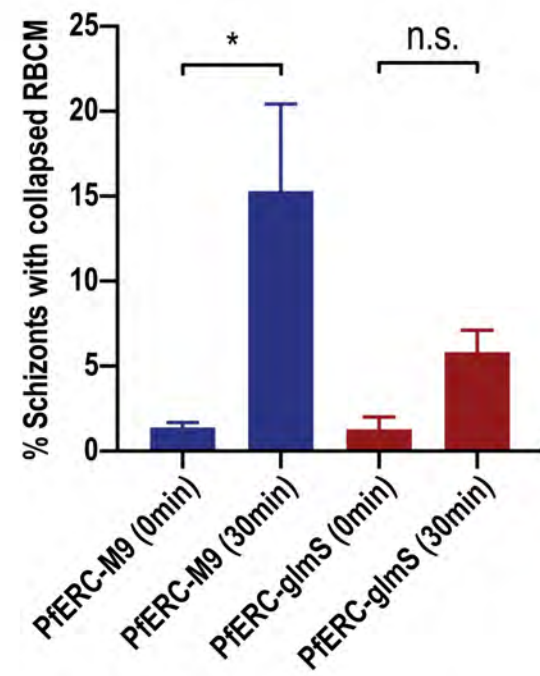

C

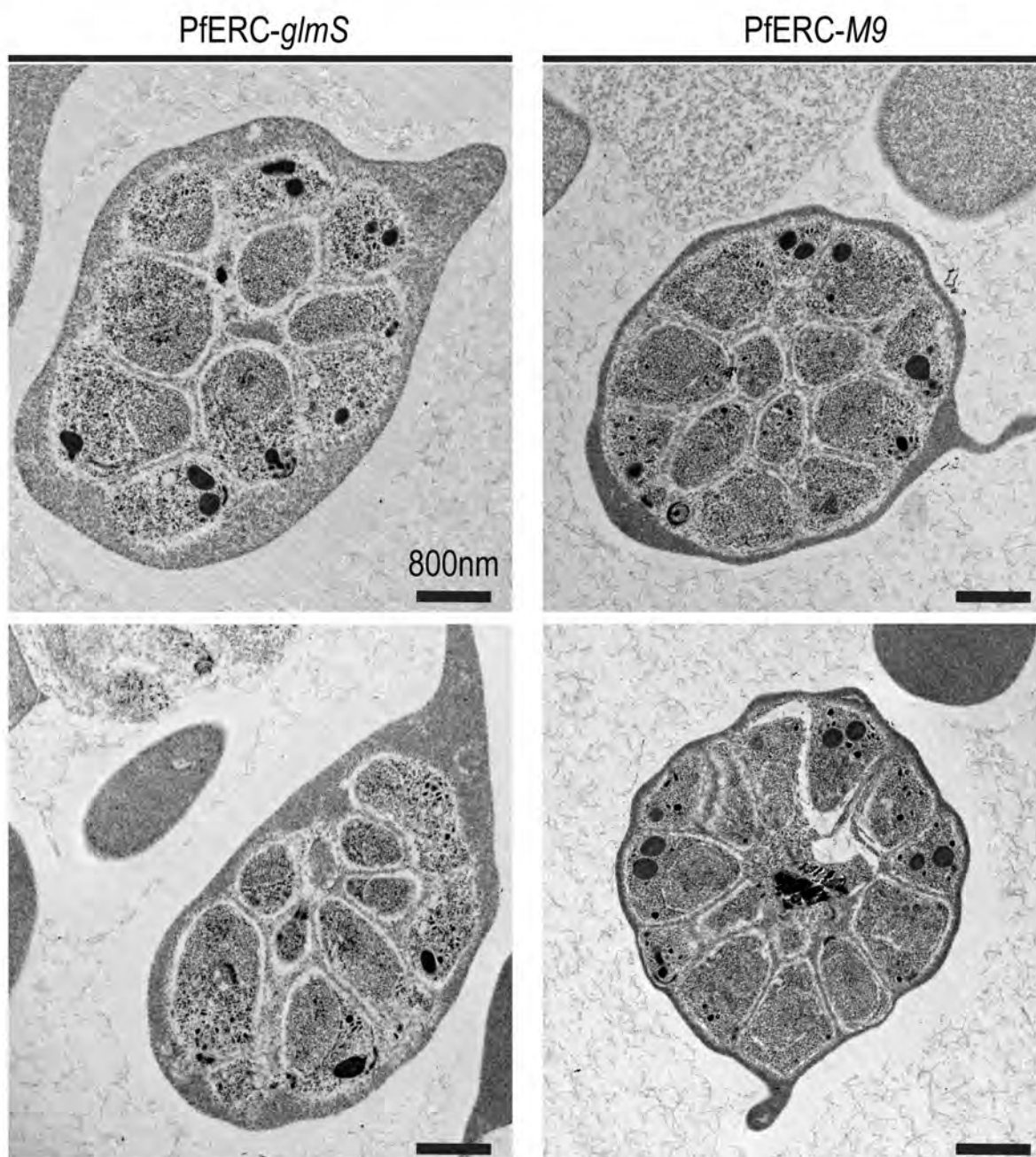

Supplement: FIG S3 [file mBio.03078-19-sf003.pdf]

**A**Saponin Isolated  
Schizonts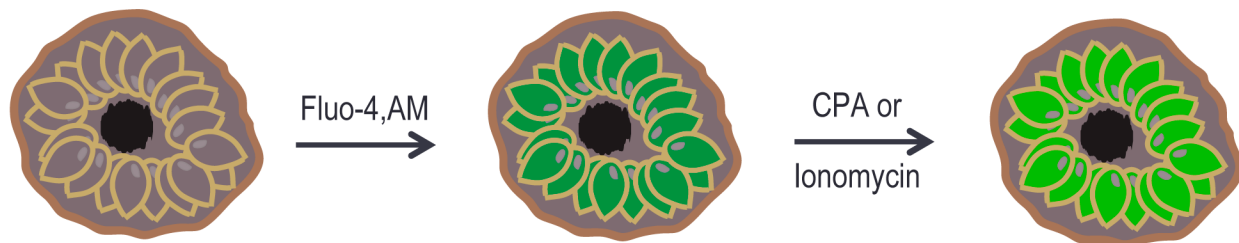**B**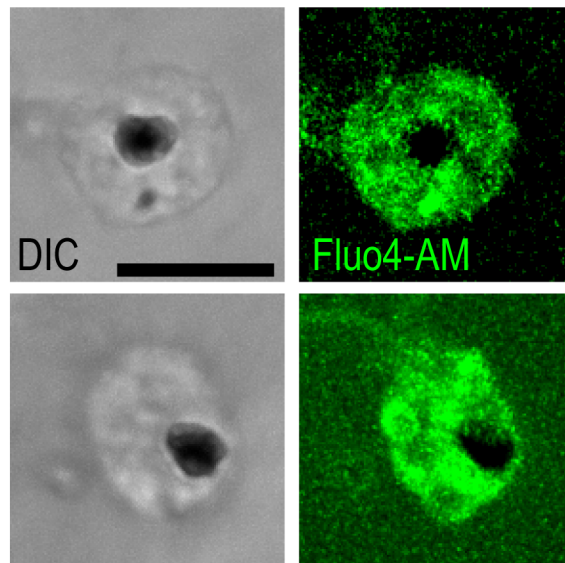**C**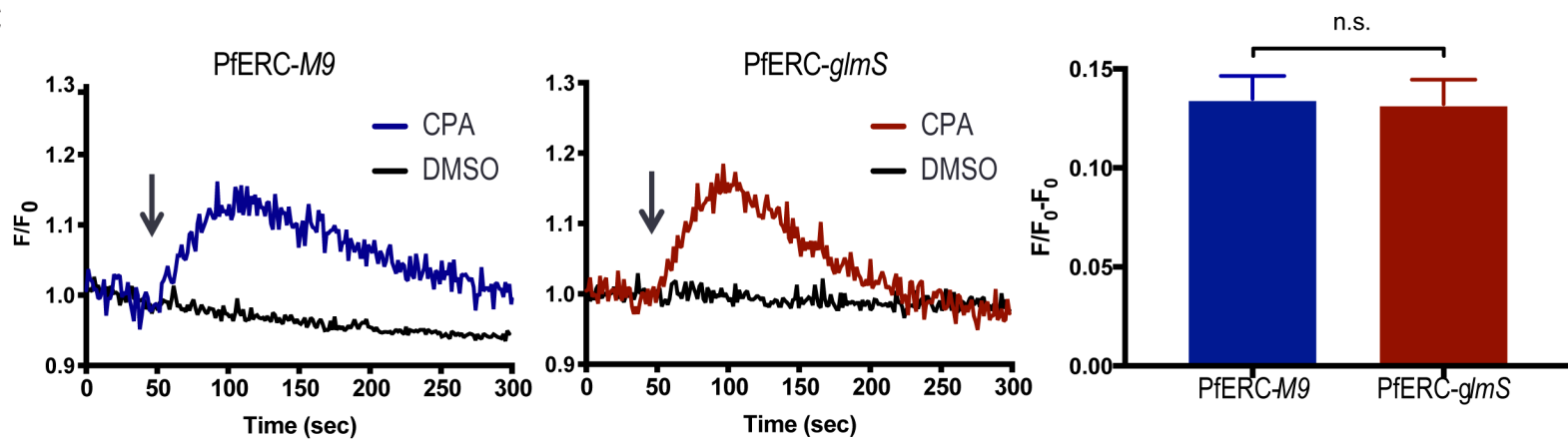**D**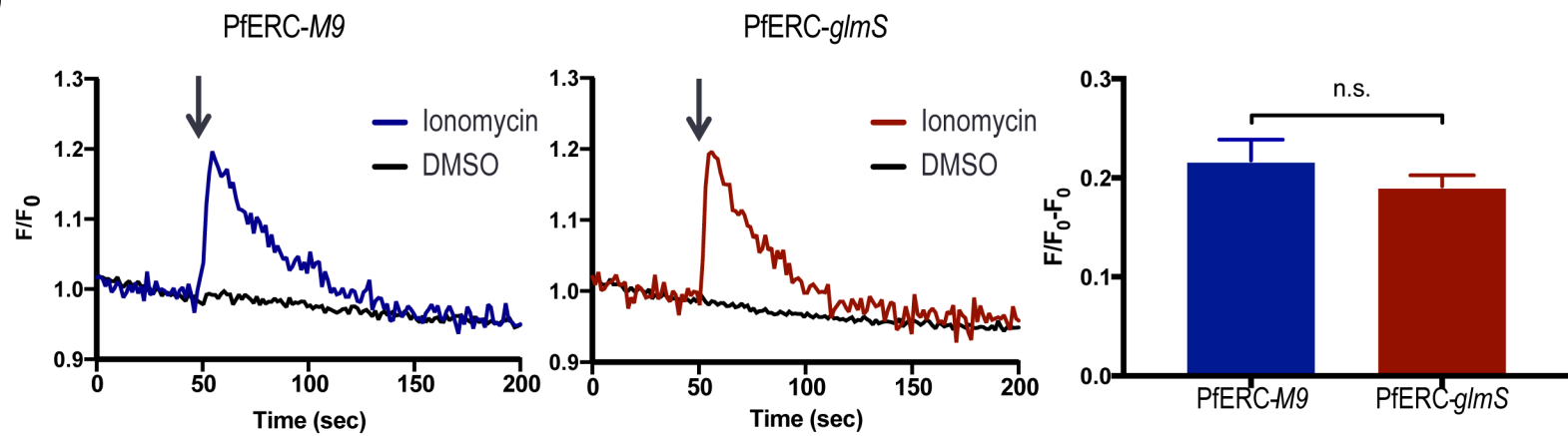

Supplement: FIG S4 [file mBio.03078-19-sf004.pdf]

PfERC-*glmS*

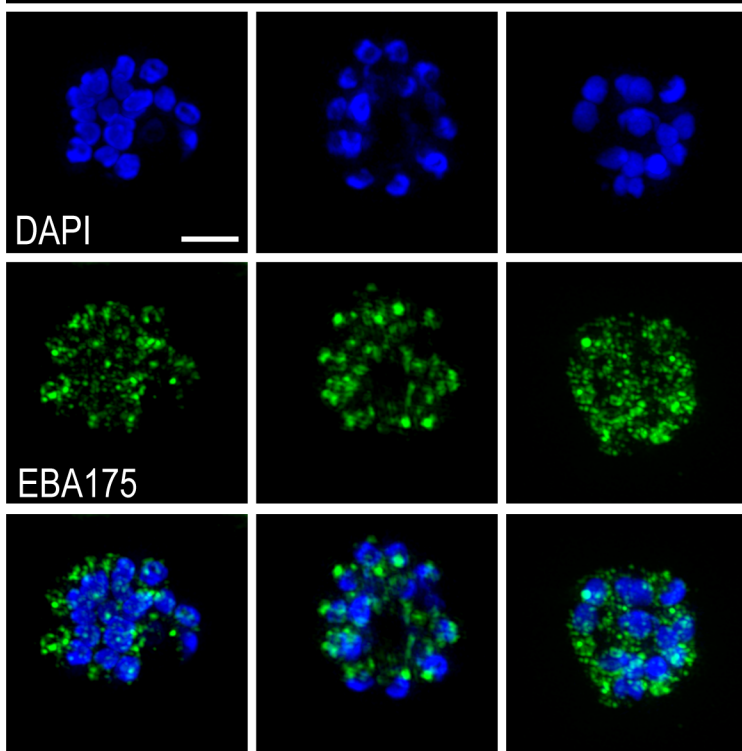

PfERC-*M9*

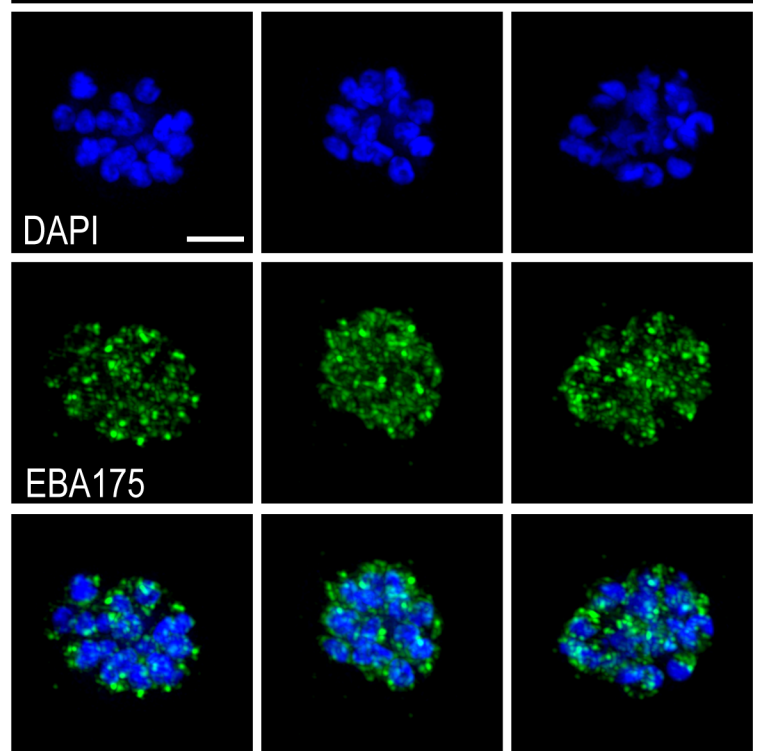

Supplement: FIG S5 [file mBio.03078-19-sf005.pdf]

**A**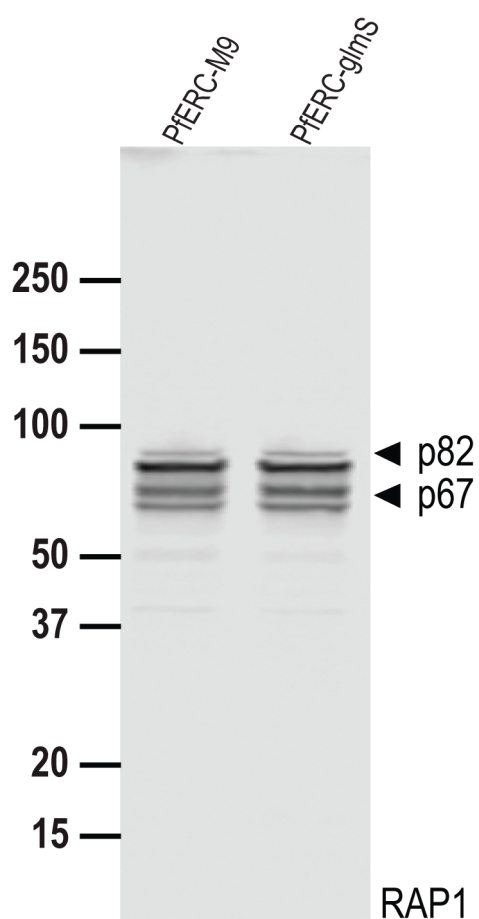**B**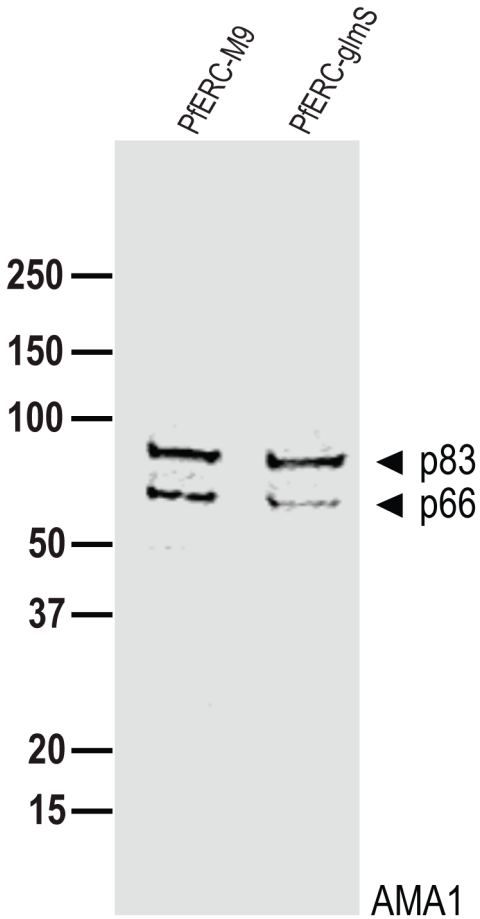**C**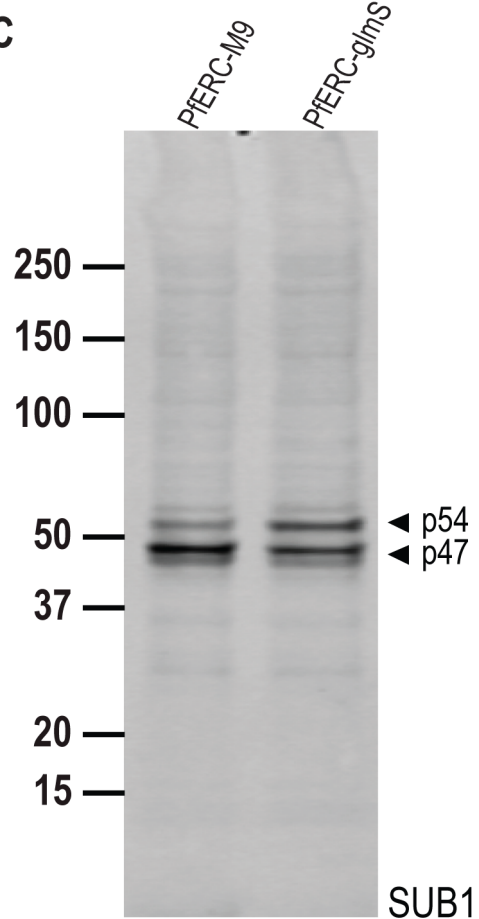**D**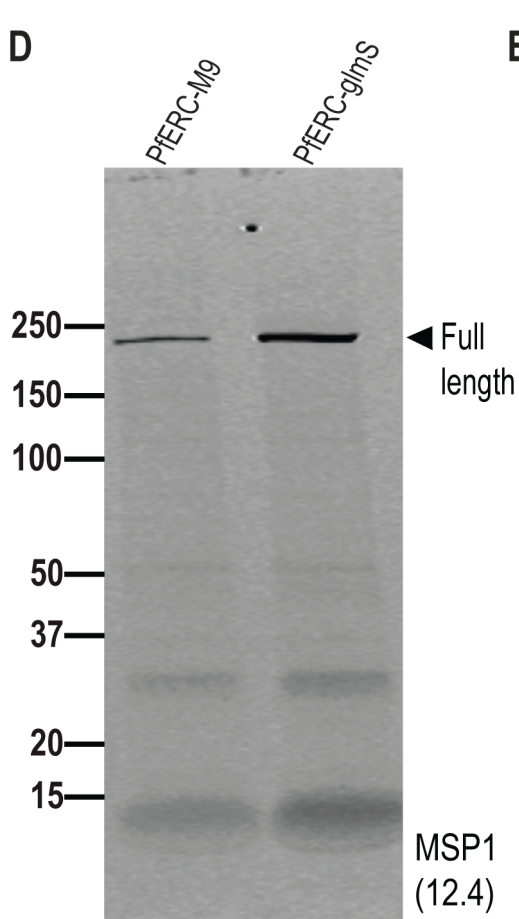**E**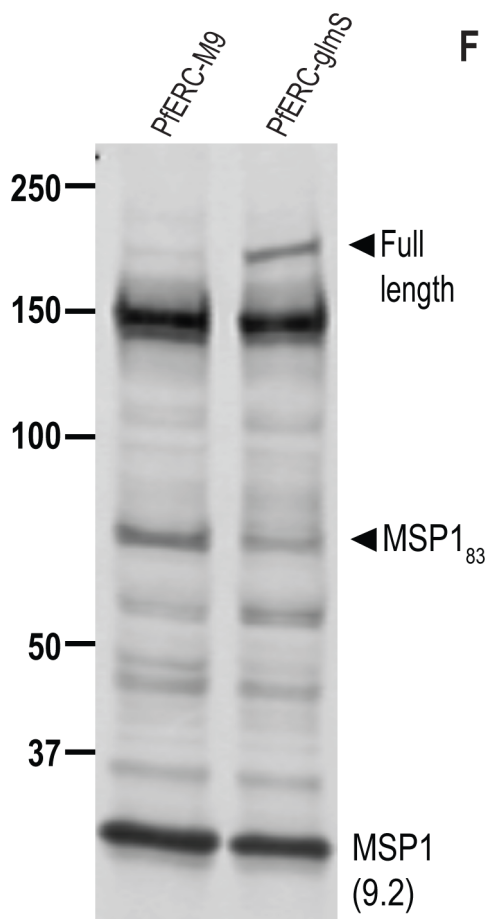**F**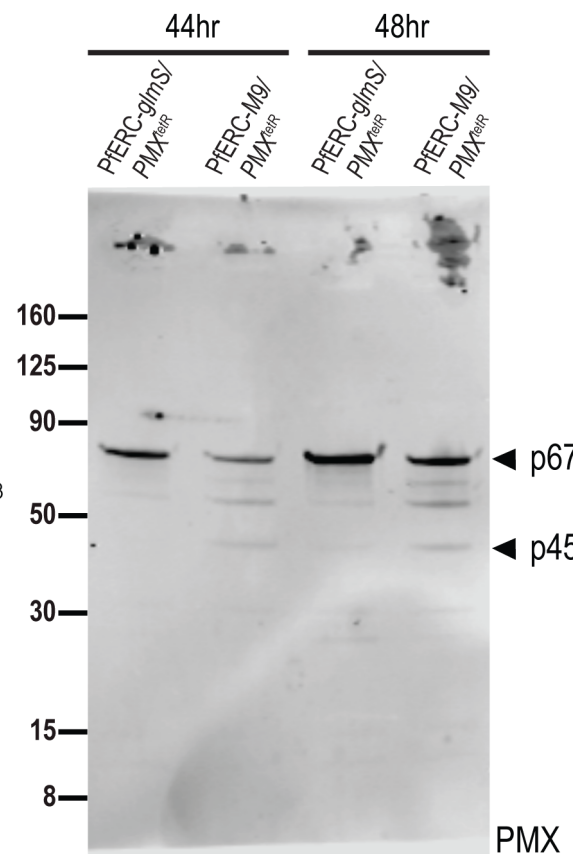

Supplement: FIG S6 [file mBio.03078-19-sf006.pdf]

A

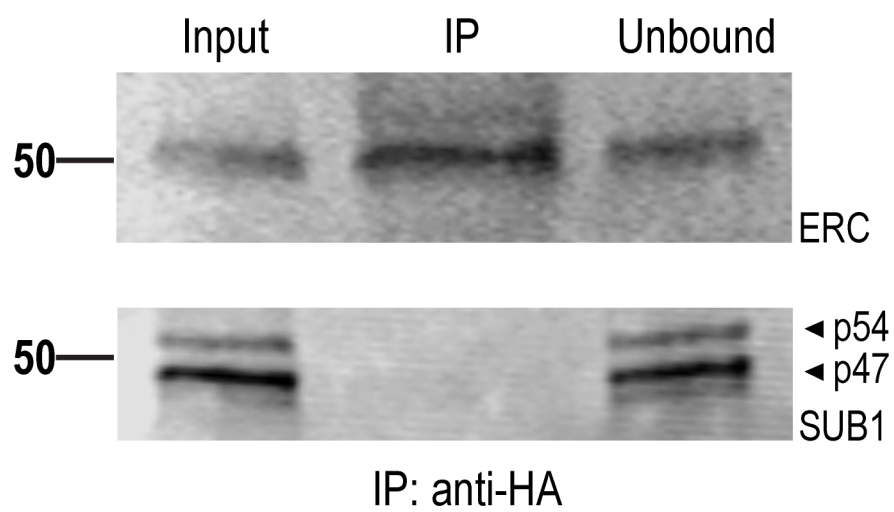

B

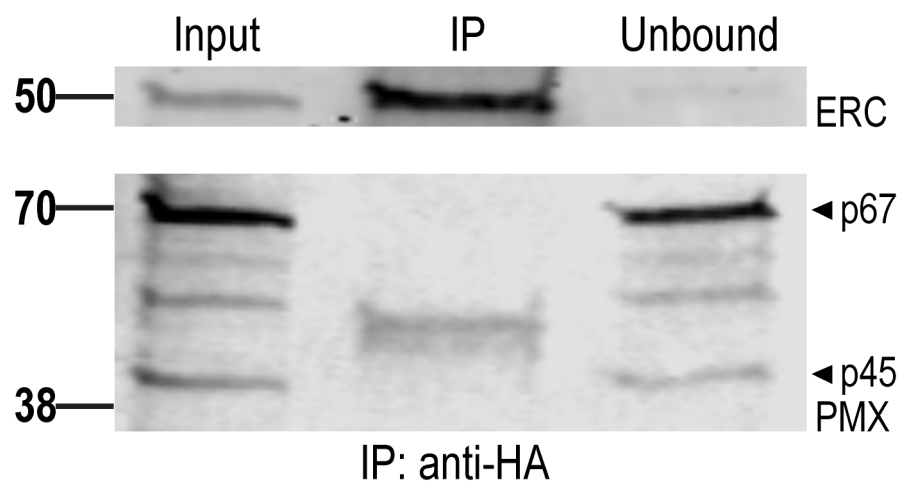

Supplement: FIG S7 [file mBio.03078-19-sf007.pdf]

**A**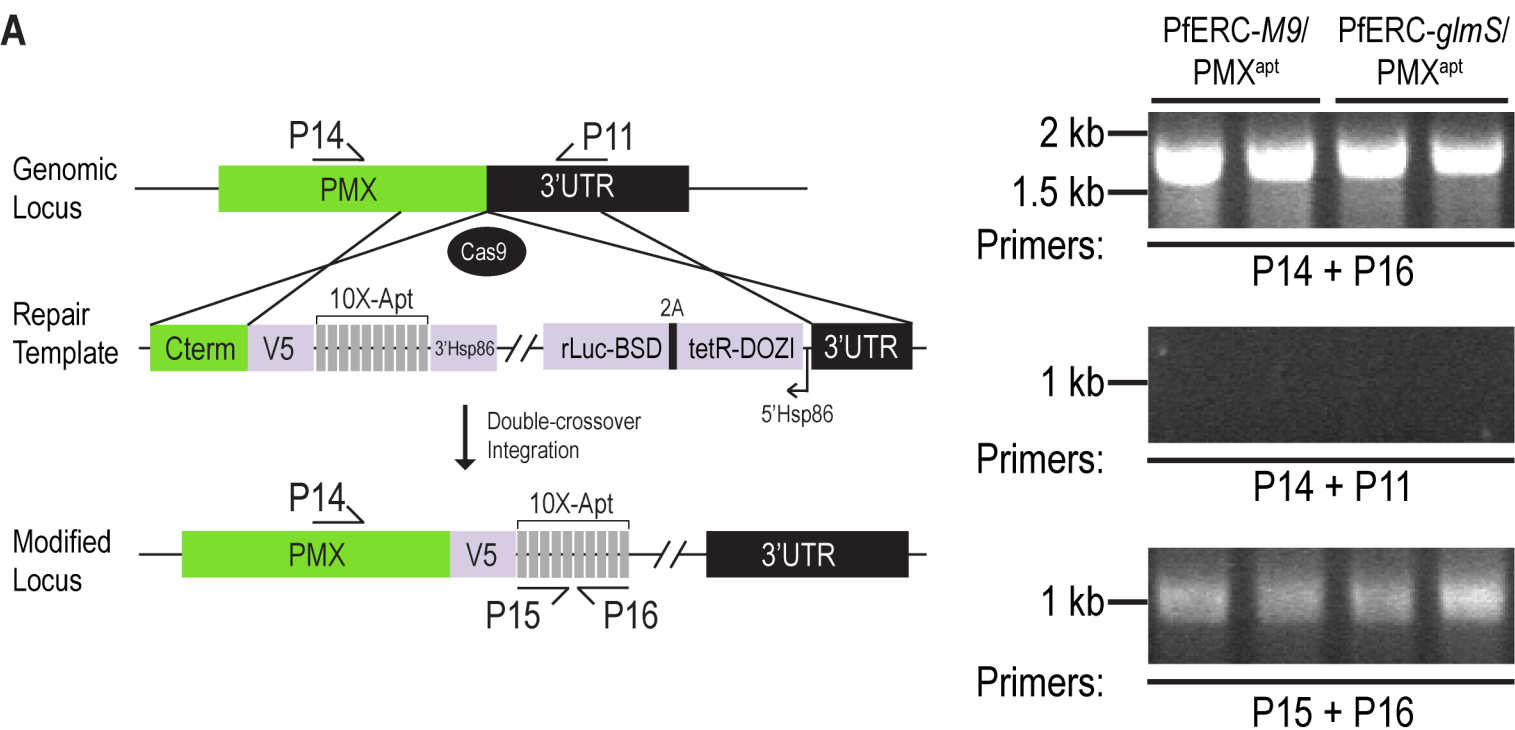**B**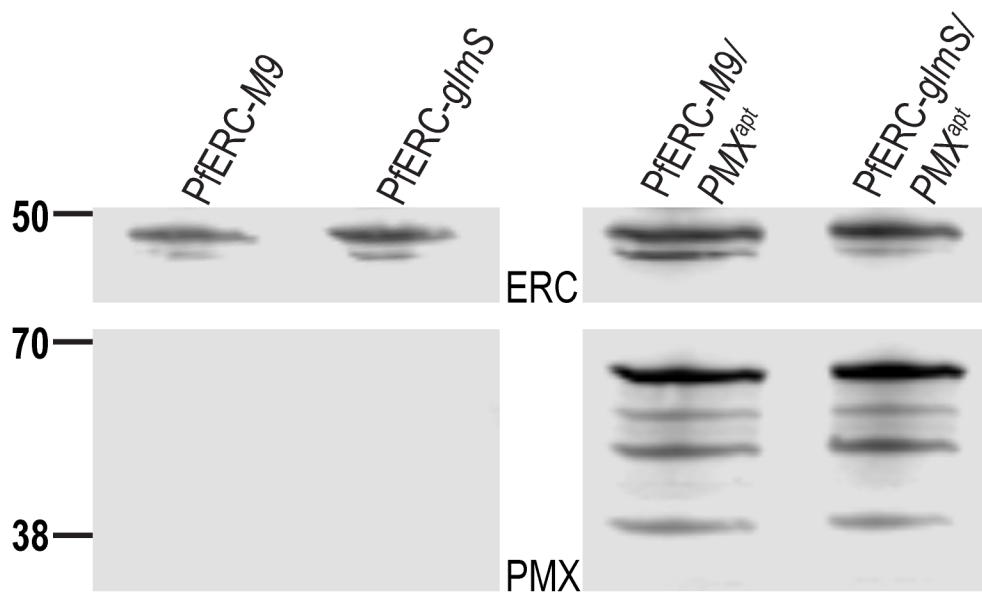

Supplement: FIG S8 [file mBio.03078-19-sf008.pdf]

PfERC-*glmS*

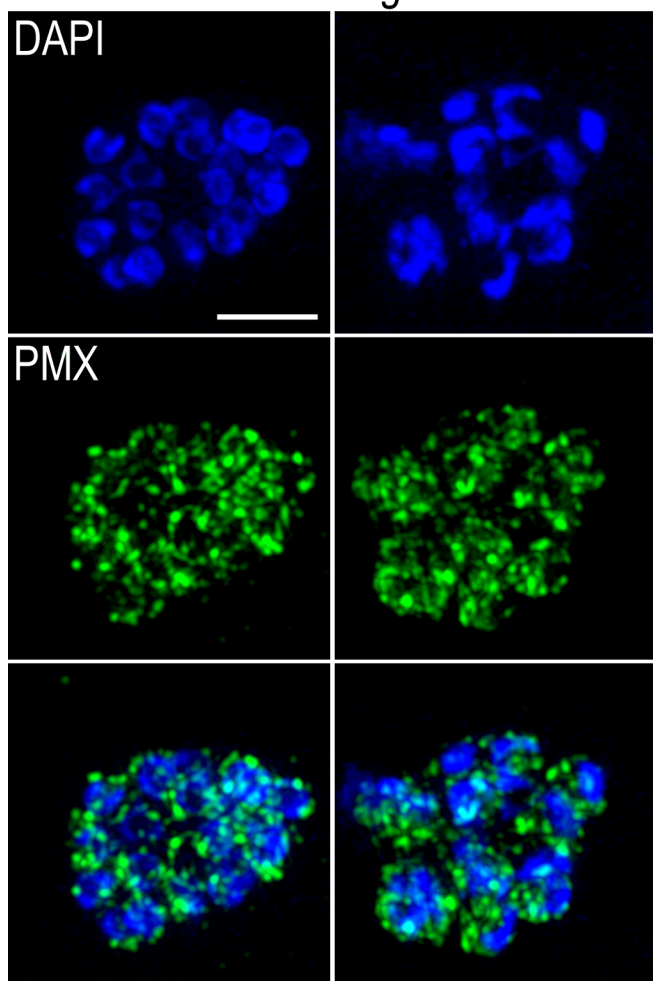

PfERC-M9

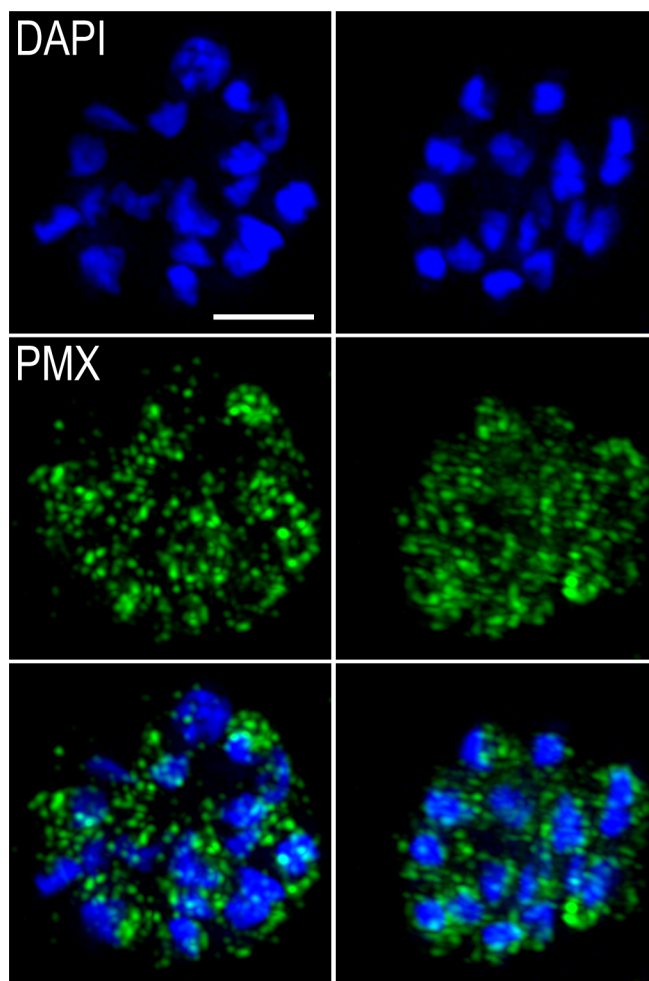

Supplement: FIG S9 [file mBio.03078-19-sf009.pdf]
